# Plasma cell-free DNA 5-hydroxymethylcytosine and whole-genome sequencing signatures for early detection of esophageal cancer

**DOI:** 10.1038/s41419-023-06329-3

**Published:** 2023-12-19

**Authors:** Di Lu, Xuanzhen Wu, Wendy Wu, Shuangxiu Wu, Hui Li, Yuhong Zhang, Xuebin Yan, Jianxue Zhai, Xiaoying Dong, Siyang Feng, Xueying Zhang, Fuming Sun, Shaobo Wang, Kaican Cai

**Affiliations:** 1 Department of Thoracic Surgery, Nanfang Hospital, Southern Medical University, Guangzhou, 510515 China; 2 Berry Oncology Corporation, Beijing, 100102 China

**Keywords:** Cancer screening, Gastrointestinal cancer, Cancer models

## Abstract

Esophageal cancer is a highly incidence and deadly disease with a poor prognosis, especially in developing countries. Owing to the lack of specific symptoms and early diagnostic biomarkers, most patients are diagnosed with advanced disease, leading to a 5-year survival rate of less than 15%. Early (*n* = 50) and middle-advanced (*n* = 50) esophageal squamous cell carcinoma (ESCC) patients, as well as 71 healthy individuals, underwent 5-hydroxymethylcytosine (5hmC) sequencing on their plasma cell-free DNA (cfDNA). A Northern Chinese cohort of cfDNA 5hmC dataset of 150 ESCC patients and 183 healthy individuals were downloaded for validation. A diagnostic model was developed using cfDNA 5hmC signatures and then improved by low-pass whole genome sequencing (WGS) features of cfDNA. Conserved cfDNA 5hmC modification motifs were observed in the two independent ESCC cohorts. The diagnostic model with 5hmC features achieved an AUC of 0.810 and 0.862 in the Southern and Northern cohorts, respectively, with sensitivities of 69.3–74.3% and specificities of 82.4–90.7%. The performance was well maintained in Stage I to Stage IV, with accuracy of 70–100%, but low in Stage 0, 33.3%. Low-pass WGS of cfDNA improved the AUC to 0.934 with a sensitivity of 82.4%, a specificity of 88.2%, and an accuracy of 84.3%, particularly significantly in Stage 0, with an accuracy up to 80%. 5hmC and WGS could efficiently differentiate very early ESCC from healthy individuals. These findings imply a non-invasive and convenient method for ESCC detection when clinical treatments are available and may eventually prolong survival.

## Introduction

Esophageal cancer (EC) is a global problem that threatens people’s health and life expectancy worldwide [1, 2]. Esophageal cancer subtypes, esophageal squamous cell carcinoma (ESCC) and esophageal adenocarcinoma (EAC), displayed distinct geographic variations [2, 3]. ESCC is apparently higher in China and in transitioning countries of Central Asia, East and South of Africa [2, 3]. Due to the absence of specific symptoms, most ESCC is found at advanced stage, limiting the clinical benefit of patients [4]. Therefore, early detection of ESCC when clinical treatments are available is an important way to prolong survival. Endoscopy is currently used for esophageal cancer diagnosis and treatment, or ESCC early detection in high-risk individuals [5, 6]. However, due to the invasive, inconvenient, time-consuming process, and low cost-effective for individuals aged <55 years, endoscopy is not suitable for large-scale screening [7, 8]. Although tumor markers such as squamous cell carcinoma antigen (SCC), carcinoembryonic antigen (CEA), and carbohydrate antigen 19-9 (CA19-9) are correlated with esophageal carcinogenesis, the sensitivity is less than 42% [9, 10]. Thus, there is an urgent demand for a less invasive, convenient, and widely available method for ESCC screening.

Liquid biopsy refers to a non-invasive and easily repeatable method that performed molecular and genic analysis of circulating tumor cells (CTCs) or cell-free DNA/RNA (cfDNA/RNA) from liquid specimens, becoming a valuable tool for cancer screening [11, 12]. However, due to the low detection rate and specificity of CTCs and the structural instability of cfRNA [13, 14], cfDNA is the most referred liquid biopsy analyte and has proved to be an approved biomarker in EC screening, detection and monitoring [15]. 5-hydroxymethylcytosine (5hmC) is recognized as a better biomarker to detect gene expression and exhibit more tissue specificity [16]. 5hmC has been used as a promising marker for cancers like early-stage pancreatic cancer, non-small-cell lung cancer (NSCLC), hepatocellular carcinoma (HCC), blood and colon cancer [17–22]. Recently, Chen et al. acquired cfDNA signatures such as shorter fragment size, special motif, and nucleosome footprint (NF) through whole genome sequencing (WGS) and identified cancers from healthy control accurately, providing a new method for cancer screening [23]. Furthermore, WGS enables the detection of variants in cancer-related genes and establishment of a comprehensive picture of the tumor in comparison with whole exon sequencing (WES) and polymerase chain reaction (PCR) while being the most rapid and cost-effective method used for cancer carrier screening [15, 24].

Therefore, we performed low-pass WGS and 5hmC technology on cfDNAs from all enrolled participants to acquire 5’ end motif, NF, fragment and 5’ hmC signatures profiles, and constructed a weighted diagnostic model based on the performance of these features to identify ESCC from healthy people in this prospective study.

## Methods

### Study participants and clinical features

All participants (aged 50–70) including ESCC (Stage 0/I, *n* = 50, stage II/III/IV, *n* = 50) and healthy control (HC) (*n* = 71) were enrolled respectively from Nanfang Hospital of Southern Medical University, with a male-to-female gender ratio of 3:1. ESCC participants were restricted to patients who has received initial treatments and were diagnosed with esophagus and esophagogastric junction cancer in accordance with the eighth edition of the AJCC/UICC cancer staging manuals at stage 0 through IV [25], and confirmed cytopathologically and histologically. Hazard covariates like living habits (especially hot food preference), family disease history, BMI, smoking, drinking, etc., were kept consistent. Participants without ESCC and other relevant diseases from the health examine center of Nanfang Hospital were selected as HC. The other criteria were consistent with ESCC group (ClinicalTrials.gov identifier, NCT03922230). Participants with insufficient data, sample contamination, or any other factors leading to termination of the study were excluded.

### Sample size calculation

Sample size calculation was referred to Hajian-Tilaki [26]. The required sample size for each group of healthy control and ESCC is defined by:

$$N=\frac{{Z}_{\frac{\alpha }{2}}^{2}V(\widehat{{AUC}})}{{d}^{2}}$$

where *α* can be caculated as follows and *φ*^−1^ is the inverse of standard cumulative normal distribution (suppose the pre-determined value of AUC = 0.934):

$$\alpha ={\varphi }^{-1}\left(0.934\right)\times 1.414=1.5153\,\times\, 1.414=2.142634$$

the $V(\widehat{{AUC}})$ can be driven as follows:

$$\begin{array}{c}{V\left(\widehat{{AUC}}\right)=\left(0.0099\times {e}^{\frac{-{\alpha }^{2}}{2}}\right)\times \left(6{\alpha }^{2}+16\right)}\\{=(0.0099\times {e}^{-\frac{{2.142634}^{2}}{2}})\times (6\times {2.142634}^{2}+16)=0.043419}\end{array}$$

In order to estimate AUC with 95% confidence the degree of precision of estimate about 0.05, the required sample size is obtained by inserting the $V(\widehat{{AUC}})$ and *d* = 0.05 as follows:

$$N=\frac{{1.96}^{2}\times 0.043419}{{0.05}^{2}}=67$$

which means there were 67 * 2 = 134 samples needed for this study.

### Blood sample preparation and cfDNA extraction

Peripheral blood samples were stored in cell-free tubes (Streck, USA) at 4 °C for no more than 72 h before being separated into plasma. Plasma cell-free DNA (cfDNA) was isolated using the MagMAX Cell-Free DNA Isolation Kit (Thermo, USA) and quantified by Qubit® 4.0 Fluorometer (Life Technologies, USA), and then the DNA fragment size composition was assayed by Fragment Analyzer (Agilent, USA).

### 5hmC sequencing and data processing

#### 5hmC library construction and sequencing

5hmC library construction was performed according to the method previously described [27]. Briefly, 5–20 ng cfDNA were end-repaired, A tailed (5X ER/A-Tailing Enzyme Mix, Enzymatics, USA) and ligated with T-adaptors on both ends (WGS Ligase, Enzymatics, USA). T-adaptors were conventional TruSeq DNA unique dual index adaptors for the illumina sequencers. The sequences of the adaptors were as follows: Index1 (i7) Adapters GATCGGAAGAGCACACGTCTGAACTCCAGTCAC [i7]ATCTCGTATGCCGTCTTCTGCTTG, Index 2 (i5) Adapters AATGATACGGCG

ACCACCGAGATCTACAC[i5]ACACTCTTTCCCTACACGACGCTCTTCCGATCT. Subsequently, ligated DNA was incubated in a 25 μl solution containing 50 mM HEPES buffer (pH = 8.0), 25 mM MgCl_2_, 60 μM UDP-6-N3-Glc (Active Motif, USA) and 12.5 U βGT (Thermo, USA) for 2 h at 37 °C. Then, 2.5 μl DBCO-PEG4-biotin (Click Chemistry Tools, USA) was added to the reaction mixture and incubated for 2 h at 37 °C. Then, the DNA was purified after the ligation with AMPure XP beads and was resuspended in Elution buffer (Qiagen,19086). The purified DNA was incubated with 0.5 μl M270 Streptavidin beads (Life Technologies, USA) pre-blocked with 0.67 mg/mL salmon sperm DNA in buffer 1 (5 mM Tris pH 7.5, 0.5 mM EDTA, 1 M NaCl and 0.2% Tween 20) for 30 min. The beads were shifted into the amplification reaction after washed. Afterwards, DNA fragments containing 5hmC features were subjected to PCR amplification, followed by the purification of the PCR products using AMPure XP beads according to the manufacturer’s instructions. Finally, sequenced on Illumina CN500. The whole process of cfDNA extraction, library construction and sequencing was blinded to the investigators, except for the sample ID.

After removed adaptor and end sequence by trim_galore software (https://github.com/FelixKrueger/TrimGalore) [28]. Acquired clean data was aligned to the human reference genome (hg19/GRCh37) by Bowtie2 v2.2.5 (http://bowtiebio.sourceforge.net/bowtie2/index.shtml) [29]. Picard Tools (http://broadinstitute.github.io/picard/) and SAMtools (https://github.com/samtools/samtools/releases/download/) [30] were used to process and filter PCR duplicates for mapped BAM files. Reads with a duplicate ratio of less than 65% and an enrichment efficiency over 95-fold were used for further analysis.

#### 5hmC peak identification

5hmC-enriched regions were identified by ChIP-seq [31] using a *q* value cut-off of 0.01 and model fold of [5, 32]). Peaks with *q* < 1E-12 and fold enrichment >8 were considered highly reliable 5hmC-enriched peaks. The 5hmC enrichment level was expressed as fragments per kilobase of 5hmC-DNA per million fragments mapped (FPKM) and the peak regions were annotated using annotatr [33]. The genome-wide distribution of 5hmC and the metagene profile were visualized using Integrated Genomics Viewer [34, 35] and ngsplot [36].

### Differential 5hmC peak regions detection

Differential 5hmC peak regions between the HC and ESCC groups were identified using DESeq2 [37]. De novo motif analysis among the differential 5hmC peaks was performed using HOMER. Functional gene ontology (GO) and Kyoto Encyclopedia of Genes and Genomes (KEGG) pathway analyses were performed using Metascape [38].

### 5hmC biomarkers identification and performance evaluation

All samples were randomly separated into the training and test set via python random. 5hmC candidate biomarkers were identified and optimized based on the Wilcoxon rank-sum test (*P* values < 0.001) and Recursive Feature Elimination—Cross Validation approach in the training set. To validate our results, the remaining samples in each group and 150 esophageal cancer and 183 healthy control plasma-5hmC data download from a published article (designated as Northern ESCC cohort) [18] were used as the internal and external test set, respectively.

Hierarchical clustering analysis of selected differential 5hmC biomarkers was visualized using R [39]. GridSearchCV was performed in conjunction with cross-validation to obtain optimal parameters for the Support Vector Machine (SVM) to ensure the model performs well. The defined 5hmC-DNA regions and their corresponding genes were finally applied to classify the test set samples.

### Low-pass whole genome sequencing and data processing

#### WGS library construction, sequencing, and quality control

After being extracted from peripheral blood, 1–10 ng cfDNA were end-repaired, ligated with T-adaptors (Berry, China). The pre-libraries were purified by Clean NGS beads (VdoBiotech, China) quantified by the KAPA Library Quantification Kit (Kapa Biosystems, USA). Subsequently, the size of cfDNA fragment was confirmed using Bioanalyzer (Agilent, USA) and was then followed by library construction. The sequencing libraries were pooled in equal amount and then sequenced on Illumina CN500 (Illumina, San Diego, USA) with an average coverage of 2× at Berry Oncology. The whole process of cfDNA extraction, library construction and sequencing was blinded to the investigators, except for the sample ID.

After the adaptor and end sequence were removed by fastp software (https://github.com/OpenGene/fastp). Acquired clean data were aligned to the human reference genome (hg19/GRCh37) using bwa-mem (https://github.com/lh3/bwa). SAMtools (http://samtools.sourceforge.net/) [30] were used to get rid of marked duplicates, unmapped reads, and low-quality reads. Only reads with a duplicate rate of less than 15% and a mapping rate of more than 95% were used for further analysis.

#### WGS-based biomarkers identification and integrated model construction

To select more effective biomarkers for distinguishing ESCC samples from healthy controls, all samples were randomly separated into the training set and the test set using python random. Wilcoxon rank-sum test was used to compare biomarker features between ESCC and HC groups. The Least Absolute Shrinkage and Selection Operator (LASSO) methods were applied to further reduce the number of biomarkers in the training set. The detailed selection process is as follows.

##### 5′ end Motif

Different types of 4mer 5′ end motif were identified using Pysam without considering chromosome Y and unidentifiable bases and then filtered out by 1) *P* ≥ 0.05 in the Wilcoxon rank-sum test; and 2) having a weight of 0 via LASSO. Eventually, 120 motif types were left for further analysis.

##### NF

A total of 30,588 genes were recruited and filtered out by 1) more than 10% of the samples had an NF score of 0 (NF score is calculated as: NF Score = (background1 + background2)/2-Promotor); 2) *P* ≥ 0.001 in the Wilcoxon rank-sum test; and 3) a weight of 0 via LASSO. Eventually, 170 genes were selected for further analysis.

##### Fragment

The whole genome, except the Y, was divided into bins of 1 M, resulting in 3055 areas. The areas with a weight of 0 were filtered out using LASSO, and finally, 10 areas were retained.

Thereafter, SVM was utilized for model construction. Tenfold cross-validation was applied to optimize the combination parameters in the training set, and the cut-off value was set at the point with the best diagnostic accuracy. To obtain the best diagnostic model, a logistic regression model was generated using the predictive score of the four individual models, which was calculated as follows.

Logistic Score = exp(Z)/(1 + exp(Z)), where *Z* = −2.57 + (3.35 × NF) + (0.05 × Fragment) + (0.75 × Motif) + (1.74 × 5hmC)

Receiver operating characteristic (ROC) curves [40] were generated to evaluate the performance of the prediction algorithm using the pROC [41] library in R. The sensitivity and specificity were estimated at the score cut-off that maximizes the sum of sensitivity and specificity, using the ROCR library in R.

## Results

### Samples composition and study design

**Fig. 1:**
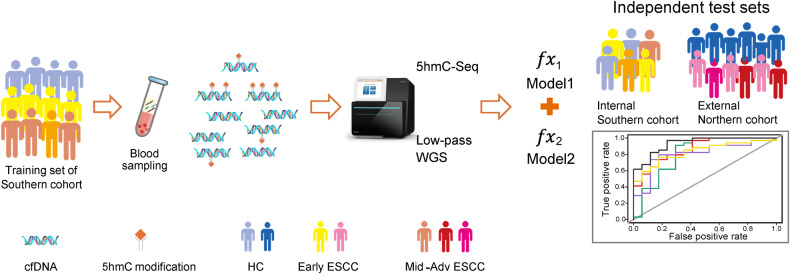
Sketch map of study design and research pipeline for early detection of ESCC. A 5hmC-based diagnostic model and low-pass WGS-based diagnostic model were developed to identify ctDNA from plasma cfDNA using a machine learning approach. A total of 171 subjects were involved as a Southern ESCC cohort, and blood samples were collected to perform 5hmC-seqeuncing and low-pass WGS, respectively. Two-thirds of the subjects were randomly selected as a training set, and the remaining one-third of the subjects were used as an independently internal Southern-ESCC test set to evaluate the model performance. A downloaded ESCC-5hmC dataset was used as an independent external Northern-ESCC test set. The research pipeline details are illustrated in supplementary Fig. S2. ctDNA cell-free tumor DNA, cfDNA cell-free DNA, HC healthy controls individuals, ESCC esophageal squamous cell carcinoma, Mid-Ad middle-advanced, 5hmC 5-hydroxymethylcytosines, WGS whole genome sequencing.

A total of 171 adult subjects, including patients with Early ESCC (stages 0, IA and IB, *n* = 50), and middle and advanced (Mid-Ad) ESCC (stages II, III and IVA, n = 50), as well as HC (*n* = 71) were prospectively enrolled from August 2018 to December 2020 from Nanfang Hospital of Southern Medical University as a Southern Chinese cohort. As illustrated in Table 1, there were no apparent differences among the clinical information, such as gender, age, BMI, living habits, differentiation of cancer, TNM stages and surgery selections, and the incidences of hypertension and diabetes (*P* > 0.05), except for family history (*P* < 0.05), which suggested that family history might play an important role in tumorigenesis. We further analyzed the clinicopathological characteristics of ESCC patients and HC, found the age and gender distributions among HC, Early ESCC and Mid-Ad ESCC groups were unbiased (*P* > 0.5, Supplementary Table S1 and Fig. S1). For biomarker screening and classifier model construction, ESCC patients and HC individuals were randomly divided into two groups: 2/3 as a training set and 1/3 as an internal validation set (Fig. 1 and Supplementary Fig. S2).

**Table 1:** Summary of demographic and clinicopathological characteristics of all the participants in this study.

Characteristics		HC (*n* = 71)	Early ESCC (*n* = 50)	Mid-Ad ESCC (*n* = 50)	*P* value
Demographic					
Gender	Male	54	37	37	
	Female	17	13	13	
Age (years) (mean, range)		58 (52–63)	60 (50–70)	59 (50–70)	0.09
BMI (mean, range)		22.9 (15.2–28.7)	22.2 (17.6–29.8)	22 (16.4–27.3)	0.28
Salted foods (like/dislike)		18 (41)^a^	16 (34)	25 (25)	0.074
Smoking (yes/no)		31 (28)^a^	30 (20)	31 (19)	0.568
Drinking (yes/no)		28 (31)^a^	20 (30)	29 (21)	0.194
Fresh vegetables and fruits (like/dislike)		42 (17)^a^	38 (12)	34 (16)	0.67
Family history (with/without)		0 (59)^a^	10 (40)	6 (44)	0.002
Hypertension (with/without)		10 (49)^a^	13 (37)	7 (43)	0.275
Diabetes (with/without)		2 (57)^a^	6 (44)	5 (45)	0.235
Clinical					
Differentiation	G0	NA	27	NA	
	G1	NA	16	20	
	G2	NA	6	21	
	G3	NA	1	9	
TNM stages	0	NA	27	NA	
	IA	NA	3	NA	
	IB	NA	20	NA	
	IIA	NA	NA	19	
	IIB	NA	NA	7	
	IIIA	NA	NA	1	
	IIIB	NA	NA	19	
	IVA	NA	NA	4	
Surgery	MATHE	NA	12	NA	
	McKeown	NA	9	24	
	Sweet	NA	18	25	
	IvorLewis	NA	NA	1	
	ESD	NA	11	NA	

Patients were classified into three groups: HC, Early ESCC, and Mid-Ad ESCC.

HC healthy controls, Mid-Ad middle-advanced, ESCC esophageal squamous cell carcinoma, MATHE mediastinoscope-assisted transhiatal esophagectomy, ESD endoscopic submucosal dissection, *P* value in chi-square test or *t* test.

^a^There were 12 unknown findings.

### Conserved 5hmC modification changes and potential biomarkers for ESCC diagnosis

**Fig. 2:**
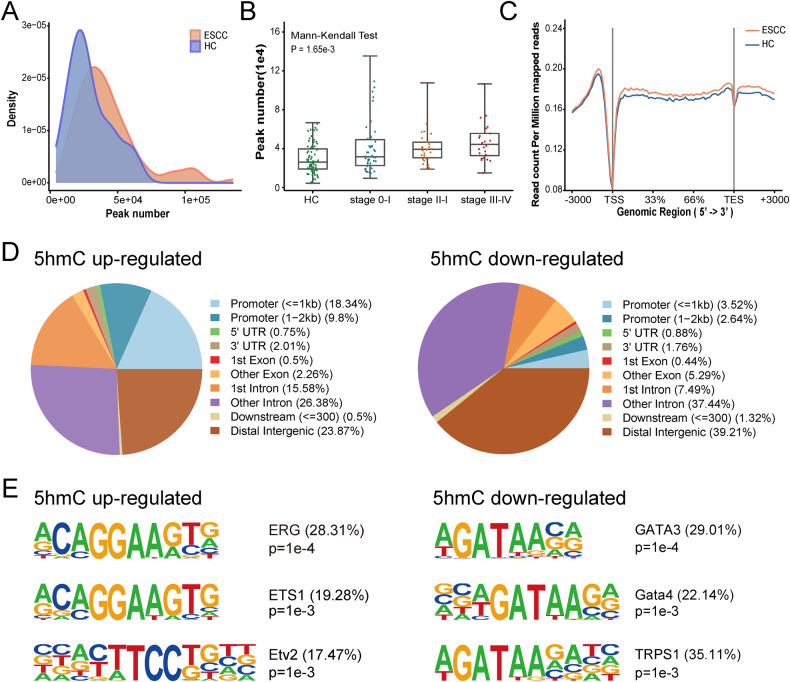
Genome-wide distribution of 5hmC signals in plasma cfDNA of ESCC and HC individuals. **A** Comparison of density distribution of 5hmC peaks number in plasma samples from 71 HC and 100 patients with ESCC. **B** Comparison of the total number of 5hmC peaks in HC and ESCC patients with stage 0–I, II, III–IV. Each dot depicts an individual cfDNA sample. *P* value shows statistical significance by Mann-Kendall Test. **C** Metagene profiles of mean values of 5hmC read counts on the regions from TSS to TES with the flanking 3000-bp in HC and ESCC samples. **D** Distribution of differential 5hmC peaks in genomic elements in ESCC samples versus HC samples. **E** Top enriched known transcription factor binding motifs detected in differential 5hmC peaks (left: 5hmC up-regulated; right: 5hmC down-regulated). Motif information was obtained from the Homer motif database. The value in parenthesis represents the percentage of target sequences enriched with the binding motif of the indicated transcription factor. HC healthy controls, ESCC esophageal squamous cell carcinoma, TSS transcription start sites, TES transcription end site, 5hmC 5-hydroxymethylcytosines.

We identified 5hmC-enriched regions in each sample and found that 5hmC peaks density exhibited a broader distribution in ESCC group compared to HC group (Fig. 2A). The number of ESCC 5hmC peaks was significantly higher than HC cohort (Mann-Whitney *U* test, *P* value = 3.11 × 10^−5^, Supplementary Fig. S3) and significantly increased from stage 0 to stage IV (Mann-Kendall Test, *P* value = 1.65 × 10^−3^, Fig. 2B). Consistent with previous study [18], the ESCC group had higher 5hmC modification levels within promoter and gene body regions (Fig. 2C). Among 398 5hmC up-regulated peaks and 227 5hmC down-regulated peaks in ESCC groups, 5hmC up-regulated peaks were significantly enriched in promoters (28.14%) and 1st intron regions (15.58%) (i.e., mainly in the regulation regions of a gene) on the whole genome level, while more 5hmC down-regulated peaks were located in other introns (37.44%) and distal intergenic regions (39.21%) (Fig. 2D).

To understand the correlation of 5hmC changes with potential binding proteins, 5hmC motif enrichment analysis was performed. Consistent with a previous study, the ERG motif (*P* = 1e-5, 28.83%) was the most significantly enriched motif in 5hmC upregulated peaks [18], followed by ETS1 (*P* = 1e-4, 20.25%) and ETV2 motif (*P* = 1e-3, 17.79%), all of which belong to the ETS transcription factors family and bind to the consensus DNA sequence 5′-AGGAA-3′ (left in Fig. 2E), most of which are downstream nuclear targets of Ras-MAP kinase signaling, and associated with cell development, differentiation, proliferation, apoptosis and tissue remodeling. In contrast, the top three motifs in 5hmC down-regulated peaks were GATA3 (*P* = 1e-5, 29.23%), GATA4 (*P* = 1e-5, 22.31%), and TRPS1 (*P* = 1e-4, 33.85%) (right in Fig. 2E). These are also consistent with previous study showing that GATA motif was identified in 5hmC-loss regions for esophageal cancer [18]. These results showed the unique signature of plasma cfDNA 5hmC, representing a potential biomarker for discriminating ESCC from healthy individuals.

### Screening, validation, and performance of candidate 5hmC biomarkers and classifier

**Fig. 3:**
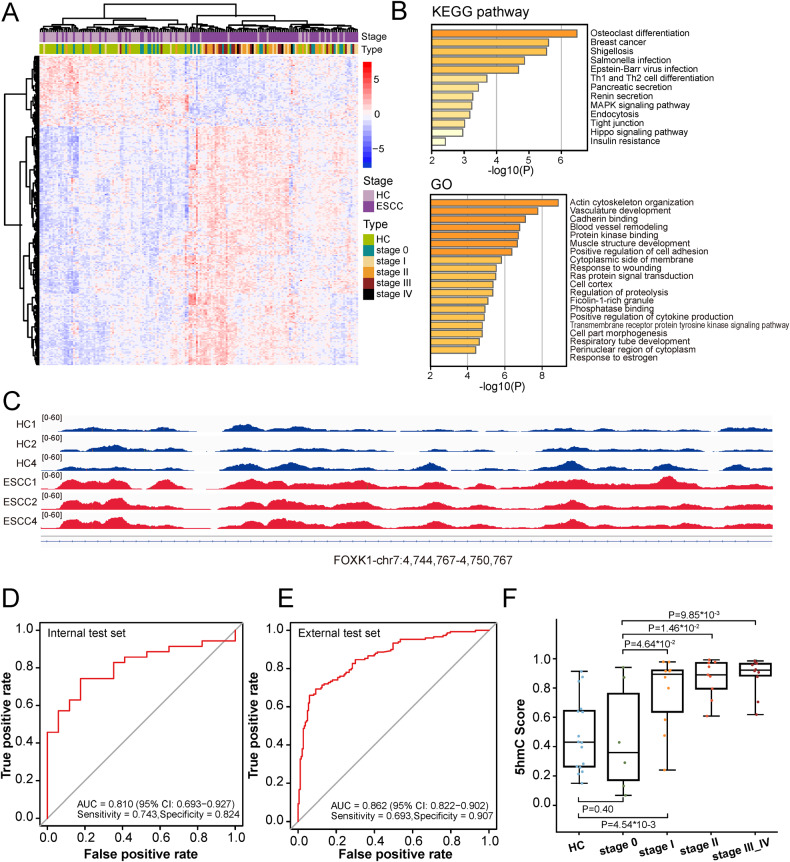
Development, validation and performance of 5hmC diagnostic model. **A** Unsupervised hierarchical clustering of 71 HC and 100 ESCC cfDNA samples based on top 273 5hmC marker genes. **B** GO enrichment (left) and KEGG pathway enrichment (right) analysis of 273 biomarkers of the 5hmC classifier. **C** The normalized 5hmC values of *FOXK1* in HC and ESCC samples. ROC curves and associated AUC values in the internal test set (**D**) and the external test set (**E**). **F** Predictive probability scores based on 5hmC classifier for different clinical stages of internal test set samples. HC healthy controls, ESCC esophageal squamous cell carcinoma, *FOXK1* forkhead box K1, ROC receiver operating characteristic, AUC area under curve, 5hmC 5-hydroxymethylcytosines.

For diagnostic model construction, 925 candidate 5hmC marker genes that derived from promoter and genebody regions were selected by Wilcoxon rank-sum test *P* values < 0.001 in the training set. Subsequently, we further identified a disease-specific panel of 273 5hmC marker genes (Supplementary Table S2), and the distinct 5hmC landscapes showed apparent separation between ESCC and HC groups (Fig. 3A). GO and KEGG analyses showed that the function of 273 5hmC biomarkers were enriched in pathways associated with cancer and metastasis and mapped to tumor-related genes (Fig. 3B). For instance, Fig. 3C exhibited the IGV plot of the high-weight biomarker located at FOXK1 gene, which plays an oncogenic role in the development of esophageal cancer [42]. 5hmC-based model illustrated decent capacity for distinguishing ESCC from HC individuals in both the internal test set (Area under curve (AUC) = 0.810 (95% CI: 0.693–0.927); sensitivity = 74.3%; specificity = 82.4%) and the external test set (AUC = 0.862 (95% CI: 0.822–0.902); sensitivity = 69.3%; specificity = 90.7%) (Fig. 3D, E). The performance in the external test set was better than the internal test set, probably caused by 27% (27/100) stage 0 patients in our cohort who might be misclassified as HC individuals.

In order to validate, we further analyzed the prediction accuracy of 5hmC biomarker classifier for different clinical stages. Though the probability of being predicted as cancer gradually increased with the progression of cancer stage (Fig. 3F), the 5hmC score between Early ESCC (stage 0 and I) and HC individuals displayed a significant disparity (*P* value = 4.35 × 10^−2^, supplementary Fig. S4), which suggested the poor capacity of the 5hmC model to discriminate Early ESCC from HC individuals. Meanwhile, the 5hmC score accurately distinguished stage I and HC samples (*P* value = 4.54 × 10^−3^), as well as stage 0 and stage I, II, III–IV (*P* values = 4.64 × 10^−2^, 1.46 × 10^−2^ and 9.85 × 10^−3^, respectively, Fig. 3F). However, in differentiating stage 0 from HC samples, 5hmC score was not the best diagnostic feature (*P* value = 0.40, Fig. 3F), and the diagnostic accuracy (33.3%, Supplementary Fig. S5B) needed to be further improved.

### Integrated model based on cfDNA signatures of low-pass WGS and 5hmC biomarkers improved diagnostic scores for early ESCC

**Fig. 4:**
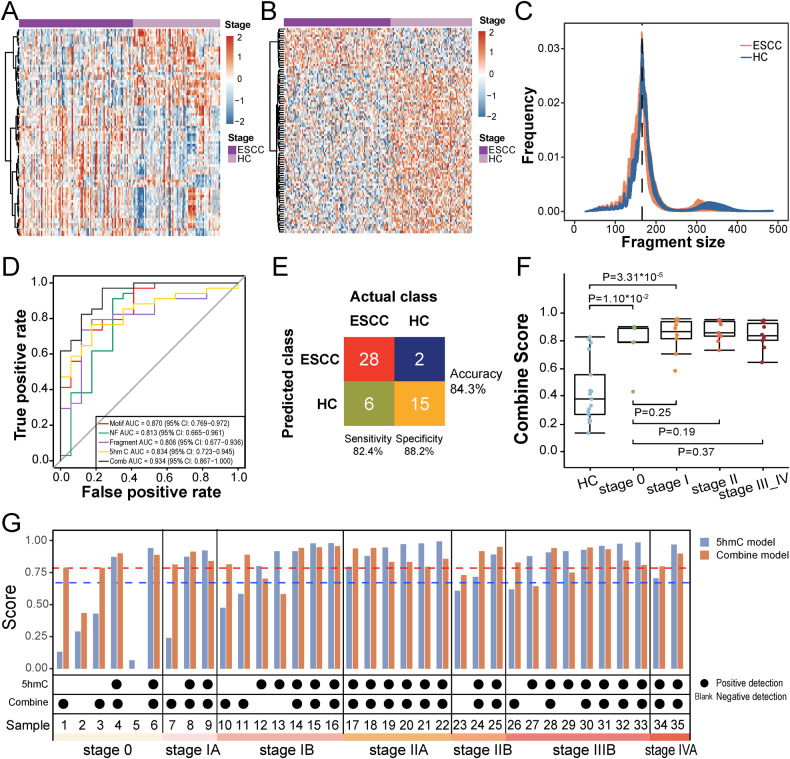
Development, validation and performance of the integrated diagnostic model. **A** Heatmap analysis of differential motifs (*p* value < 0.001) between ESCC and HC samples. **B** Heatmap analysis of genes with differential reads coverage between gene promoter and background regions (*p* values < 0.001) in ESCC and HC samples. **C** Frequencies comparison of different fragment sizes between ESCC and HC samples. **D** ROC curves and associated AUC values in the test set. **E** Confusion matrices of integrated diagnostic model comparing the actual class with the predicted class for ESCC (*n* = 34) and HC (*n* = 17) samples in the test set. **F** Predictive probability scores based on integrated diagnostic model for different clinical stages of the test set samples. **G** Comparison of diagnostic performance between 5hmC model (blue) and integrated model (red) on different clinical stages of the test set ESCC samples (*n* = 35). The blue and red dotted line represent the thresholds of diagnostic positive of the 5hmC model and the integrated model, respectively. Positive ESCC detection is indicated by black dots, negative ESCC detection indicated by blank. HC healthy controls, ESCC esophageal squamous cell carcinoma, ROC receiver operating characteristic, AUC area under curve, 5hmC 5-hydroxymethylcytosines.

To explore the prediction potential of plasma cfDNA and search for more effective biomarkers, we employed low-pass WGS to acquire genome-wide 5′ end motif [43], NF [44] and fragmentation [45] profiles from 71 HC and 93 ESCC samples. ESCC were clearly separated from HC samples by differential 5′ end motif hierarchical clustering (Fig. 4A). NF heatmap analysis indicated that genes with differential reads coverage between promoter and background regions (*P* < 0.001) held power to distinguish ESCC from HC (Fig. 4B). The cfDNA fragment size of ESCC was more variable and much shorter (median size < 150 bp) than HC (Fig. 4C). Collectively, all three genome features of cfDNA showed promising diagnostic potential for ESCC.

As illustrated in Fig. 1 and Supplementary Fig. S2, HC individuals and patients with Early and Mid-Ad ESCC were randomly assigned to a training set (about 2/3 of samples, including 54 HC, 30 Early ESCC, and 29 Mid-Ad ESCC) and a validation set (the rest of the samples). Eventually, 120 differential motif types, 170 differential NF genes and 10 fragment areas were selected for model training (Supplementary Tables S3A–S3C). The motif-based discrimination model achieved an AUC value of 0.870 (95% CI: 0.769–0.972) with sensitivity of 73.5% at specificity of 82.4% for ESCC classification in the test set (Fig. 4D, Supplementary Fig. S5A). The NF and fragmentation model achieved less powerful performance with an AUC value of 0.813 (95% CI: 0.665–0.961) and 0.806 (95% CI: 0.677–0.936), respectively (Fig. 4D). Compared with the 5′ end motif model, the fragmentation model demonstrated a higher sensitivity of 79.4% at the same specificity (82.4%), and the NF model showed excellent sensitivity of 91.2% but a lower specificity of 70.6% (Supplementary Fig. S5A).

An integrated diagnostic model was constructed by combining genomic features and 5hmC biomarkers, and it achieved an excellent AUC of 0.934 (95% CI: 0.867–1.000) with a sensitivity of 82.4%, specificity of 88.2%, and accuracy of 84.3% for ESCC classification in the test set (Fig. 4D, E). The diagnostic score showed an increasing trend from HC to ESCC, and the scores in stage 0 and I patients were significantly higher than those in HC (*P* values = 1.10 × 10^−2^ and 3.31 × 10^−5^, respectively, Fig. 4F), implying the integrated model had great potentials for ESCC early diagnosis. The integrated model had good but slightly reduced power to call stage III–IV patients (Fig. 4F), who are supposed to have more complex tumorous DNA profiles. We compared the diagnostic performance between the 5hmC model and integrated model on each ESCC patients in the test set (Fig. 4G). The integrated model displayed a higher prediction accuracy than the 5hmC model for early ESCC detection, especially in stage 0 (80.0% vs 33.3%, Fig. 4G, Supplementary Fig. S5B). For advanced ESCC patients (stage IIIB and IVA), the 5hmC model showed better performance. These data demonstrate that genome-wide integration is a sensitive and robust approach for early-stage ESCC screening.

## Discussion

Due to the absence of specific symptoms and lack of effective curable methods, ESCC is one of the most deadly cancers worldwide. ESCC screening mainly depends on endoscopy and tumor markers such as SCC, CEA and CA19-9 [8, 10]. However, invasiveness and inconvenience of the endoscopy and low sensitivity and specificity of tumor markers limited the detection of ESCC at early stage. Recently, liquid biopsy such as 5hmC and WGS were found to be potentially used in cancer screening and the sensitivity and specificity of which are up to 93.75% and 85.71%. Nonetheless, the participants either lack stage 0 or the sensitivity of stage 0 and I ESCC detection was unsatisfactory [7, 18], which means the availability of 5hmC or WGS in early ESCC detection is insufficient.

In this prospective study, we employed 5hmC and WGS on ESCC and HC participants respectively, and constructed classifiers using 5hmC biomarkers only or 5hmC combination with low-pass WGS to perform early ESCC detection. On the utilization of 5hmC markers, we distinguished ESCC patients from HC individuals in both internal and external test set. The performance of 5hmC classifier in different ESCC stages was outstanding, which is consistent with the prediction that 5hmC has the potential to be promising biomarkers for non-invasive detection of EC. Significant differences of each comparable group (Early ESCC (stage 0 and I) vs HC individuals, stage I vs HC samples, and stage 0 vs stage I/II/III–IV) implied 5hmC may participate in tumor progress and can be used in ESCC monitoring. However, even though we optimized the inclusion strategy through enrolled stage 0 ESCC patients in comparison with previous study to enhance the detection accuracy of early ESCC from healthy [46], the accuracy of differential stage 0 from HC is only 33.3%, which means to identify stage 0 from HC based on 5hmC only is difficult and need further investigation.

WGS could provide the whole genomic profile of tumor DNA and has been widely used in cancer detection, diagnosis and monitoring [47]. Recently, an integrated method based on the unique genome features of cfDNA derived from WGS for HCC diagnosis was constructed and accurately distinguish HCC from HC [27, 48]. Considering the importance of early ESCC detection and the deficient detection efficiency between early-stage ESCC (stage 0) and HC samples, we establish an integrated diagnostic classifier consisting of genome-wide 5′ end motif, NF, fragmentation profiles that derived from low pass WGS, and 5hmC biomarkers, and achieved an excellent AUC value of 0.934 with a sensitivity of 82.4%, specificity of 88.2%, and accuracy of 84.3% for ESCC patient classification in the test set. It should be noted that the diagnostic scores of ESCC patients with stage 0 and I were significantly higher than that of HC subjects (*P* values = 1.10 × 10^−2^ and 3.31 × 10^−5^, respectively). The combination of low-pass WGS cfDNA signatures and 5hmC biomarkers improved the classifier’s efficiency from 65% to 82% of sensitivity at the specificity of 88% on an overall level. Most importantly, for stage 0 patients who had low disease burden, the combined classifier significantly improved the prediction accuracy from 33.3% to 80.0%. The sensitivity of early ESCC was significantly higher than previous study [7], which suggested a better performance of early ESCC detection, especially in stage 0.

In general, more and more studies have shown that integrating multi-omics detection is a promising methodology for non-invasive early diagnosis of many types of cancer. Both 5mC and 5hmC were presumed to have an important role in gene expression and regulation, and their modification changes were observed in a wide range of malignant tumors, including ESCC [32, 49–52]. Similarly, the combination of 5hmC and WGS efficiently differentiated very early ESCC from HC either in south or north cohort, implying curable treatment and better survival of ESCC. Combining these epigenomics signal detection with whole-genome-wide features was worthy of attempts to further improving the specificity and sensitivity for early diagnosis of different subtypes and stages of ESCC patients. Although the test cohort population in this study was still limited, further investigations about the stability of this model, the discriminating capabilities for different subtypes of esophageal cancer, or the practical application values are needed to execute. The performance of 5hmC and WGS afford a non-invasive and convenient method for the early detection of ESCC. The potential utilization of multi-omics provides an innovative clinical diagnostic strategy and will ultimately bring ESCC with positive benefits.

### Supplementary information

Supplementary Materials

The reproducibility informations relevant to the study.

## Data Availability

All of the raw and processed data used in this study have been uploaded to CNGB Sequence Archive (CNSA) of China National GeneBank DataBase (CNG‑Bdb) (https://db.cngb.org/search/) with the Accession Number CNP0004480. Or you can contact the corresponding author (Kaican Cai, doc_cai@163.com) directly for data accession. The R code related to classifier detection and modeling is available upon request.
