# Supplementary material for: Plasma cell-free DNA 5-hydroxymethylcytosine and whole-genome sequencing signatures for early detection of esophageal cancer
Source: Cell Death Dis. 2023 Dec 19;14(12):843. doi: 10.1038/s41419-023-06329-3 (PMC10730877; doi:10.1038/s41419-023-06329-3)
Supplement: Supplementary file 1 — Supplementary Materials [file 41419_2023_6329_MOESM1_ESM.pdf]

Table S1A. Summary of demographic and clinicopathological characteristics of 100 esophageal cancer patients and 71 healthy controls in this study.

Characteristics		Healthy controls (n=71)	Early ESCC patients (n=50)	Mid-Ad ESCC patients (n=50)
**Demographic**				
**Age at surgery, years, Mean**		58 (52-63)	61 (55-67)	59 (55-64)
**Gender, n (%)**	Male	55 (77)	38 (76)	37 (74)
	Female	16 (23)	12 (24)	13 (26)
**Clinical**				
**TNM stage, n (%)**	0	-	27 (27)	-
	IA	-	3 (3)	-
	IB	-	20 (20)	-
	IIA	-	-	19 (19)
	IIB	-	-	7 (7)
	IIIA	-	-	1 (1)
	IIIB	-	-	19 (19)
	IV	-	-	4 (4)

Note: Mid-Ad, middle to advanced; ESCC, esophageal squamous cell carcinoma.

Table S1B. Clinicopathological characteristics of 100 esophageal cancer patients and 71 healthy controls.

Sample ID	Population	Gender	Age	Caner Subtype	TNM Stage	Subgroup
C1802961	Chinese	Male	57	Squamous carcinoma	P0	Early
C1802962	Chinese	Male	59	Squamous carcinoma	P0	Early
C1802973	Chinese	Male	69	Squamous carcinoma	P0	Early
C1802975	Chinese	Male	70	Squamous carcinoma	P0	Early
C1802996	Chinese	Male	66	Squamous carcinoma	P0	Early
C1802998	Chinese	Male	48	Squamous carcinoma	P0	Early
C1803017	Chinese	Male	55	Squamous carcinoma	P0	Early
C1803018	Chinese	Male	60	Squamous carcinoma	P0	Early
C1803019	Chinese	Male	66	Squamous carcinoma	P0	Early
C1803020	Chinese	Male	52	Squamous carcinoma	P0	Early
C1803023	Chinese	Male	67	Squamous carcinoma	P0	Early
C1803026	Chinese	Male	55	Squamous carcinoma	P0	Early
C1803050	Chinese	Male	59	Squamous carcinoma	P0	Early
C1906074	Chinese	Male	63	Squamous carcinoma	P0	Early
C1906075	Chinese	Male	68	Squamous carcinoma	P0	Early
C1803070	Chinese	Female	54	Squamous carcinoma	P0	Early
C1803071	Chinese	Male	68	Squamous carcinoma	P0	Early
C1803072	Chinese	Male	53	Squamous carcinoma	P0	Early
C1803075	Chinese	Male	63	Squamous carcinoma	P0	Early
C1803078	Chinese	Male	65	Squamous carcinoma	P0	Early
C1906095	Chinese	Female	70	Squamous carcinoma	P0	Early
C1906096	Chinese	Female	53	Squamous carcinoma	P0	Early
C2005345	Chinese	Female	70	Squamous carcinoma	P0	Early
C2005346	Chinese	Male	65	Squamous carcinoma	P0	Early
C2005347	Chinese	Female	60	Squamous carcinoma	P0	Early
C2005348	Chinese	Male	61	Squamous carcinoma	P0	Early
C2005349	Chinese	Male	68	Squamous carcinoma	P0	Early
C1802967	Chinese	Male	67	Squamous carcinoma	ⅠA	Early
C1802969	Chinese	Male	68	Squamous carcinoma	ⅠA	Early
C1803024	Chinese	Male	68	Squamous carcinoma	ⅠA	Early
C1802283	Chinese	Female	53	Squamous carcinoma	ⅠB	Early
C1802970	Chinese	Male	57	Squamous carcinoma	ⅠB	Early
C1802971	Chinese	Male	50	Squamous carcinoma	ⅠB	Early
C1802972	Chinese	Male	50	Squamous carcinoma	ⅠB	Early
C1802977	Chinese	Male	50	Squamous carcinoma	ⅠB	Early
C1802980	Chinese	Male	62	Squamous carcinoma	ⅠB	Early
C1802981	Chinese	Male	65	Squamous carcinoma	ⅠB	Early

C1802985	Chinese	Male	48	Squamous carcinoma	Ⅰ B	Early
C1802990	Chinese	Male	50	Squamous carcinoma	Ⅰ B	Early
C1802997	Chinese	Female	66	Squamous carcinoma	Ⅰ B	Early
C1803003	Chinese	Male	64	Squamous carcinoma	Ⅰ B	Early
C1803007	Chinese	Female	61	Squamous carcinoma	Ⅰ B	Early
C1803009	Chinese	Female	64	Squamous carcinoma	Ⅰ B	Early
C1803051	Chinese	Female	56	Squamous carcinoma	Ⅰ B	Early
C1803052	Chinese	Male	56	Squamous carcinoma	Ⅰ B	Early
C1803061	Chinese	Male	70	Squamous carcinoma	Ⅰ B	Early
C1803069	Chinese	Female	58	Squamous carcinoma	Ⅰ B	Early
C1803062	Chinese	Male	58	Squamous carcinoma	Ⅰ B	Early
C1803085	Chinese	Female	67	Squamous carcinoma	Ⅰ B	Early
C2005319	Chinese	Male	57	Squamous carcinoma	Ⅰ B	Early
Z18W01296	Chinese	Male	50	Squamous carcinoma	Ⅱ A	Mid-Ad
C1802960	Chinese	Male	55	Squamous carcinoma	Ⅱ A	Mid-Ad
C1802978	Chinese	Male	69	Squamous carcinoma	Ⅱ A	Mid-Ad
C1802979	Chinese	Male	51	Squamous carcinoma	Ⅱ A	Mid-Ad
C1802983	Chinese	Male	66	Squamous carcinoma	Ⅱ A	Mid-Ad
C1802988	Chinese	Male	54	Squamous carcinoma	Ⅱ A	Mid-Ad
C1802991	Chinese	Male	56	Squamous carcinoma	Ⅱ A	Mid-Ad
C1802995	Chinese	Male	57	Squamous carcinoma	Ⅱ A	Mid-Ad
C1803000	Chinese	Male	56	Squamous carcinoma	Ⅱ A	Mid-Ad
C1803002	Chinese	Male	56	Squamous carcinoma	Ⅱ A	Mid-Ad
C1803004	Chinese	Male	64	Squamous carcinoma	Ⅱ A	Mid-Ad
C1803005	Chinese	Female	64	Squamous carcinoma	Ⅱ A	Mid-Ad
C1903184	Chinese	Male	64	Squamous carcinoma	Ⅱ A	Mid-Ad
C1803016	Chinese	Male	54	Squamous carcinoma	Ⅱ A	Mid-Ad
C1803022	Chinese	Male	68	Squamous carcinoma	Ⅱ A	Mid-Ad
C1803025	Chinese	Female	60	Squamous carcinoma	Ⅱ A	Mid-Ad
C1803049	Chinese	Female	68	Squamous carcinoma	Ⅱ A	Mid-Ad
C1803077	Chinese	Female	69	Squamous carcinoma	Ⅱ A	Mid-Ad
C1906087	Chinese	Female	58	Squamous carcinoma	Ⅱ A	Mid-Ad
C1802963	Chinese	Male	55	Squamous carcinoma	Ⅱ B	Mid-Ad
C1802964	Chinese	Male	57	Squamous carcinoma	Ⅱ B	Mid-Ad
C1802965	Chinese	Male	56	Squamous carcinoma	Ⅱ B	Mid-Ad
C1802982	Chinese	Female	56	Squamous carcinoma	Ⅱ B	Mid-Ad
C1802984	Chinese	Male	53	Squamous carcinoma	Ⅱ B	Mid-Ad
C1803011	Chinese	Male	67	Squamous carcinoma	Ⅱ B	Mid-Ad
C2005343	Chinese	Female	66	Squamous carcinoma	Ⅱ B	Mid-Ad
C1803047	Chinese	Female	56	Squamous carcinoma	ⅢA	Mid-Ad
Z18W01385	Chinese	Male	52	Squamous carcinoma	ⅢB	Mid-Ad
C1803080	Chinese	Male	52	Squamous carcinoma	ⅢB	Mid-Ad

Z18W01384	Chinese	Male	60	Squamous carcinoma	ⅢB	Mid-Ad
C1802966	Chinese	Male	53	Squamous carcinoma	ⅢB	Mid-Ad
C1802968	Chinese	Male	60	Squamous carcinoma	ⅢB	Mid-Ad
C1802974	Chinese	Male	56	Squamous carcinoma	ⅢB	Mid-Ad
C1802976	Chinese	Male	56	Squamous carcinoma	ⅢB	Mid-Ad
C1802987	Chinese	Male	67	Squamous carcinoma	ⅢB	Mid-Ad
C1802989	Chinese	Male	50	Squamous carcinoma	ⅢB	Mid-Ad
C1802992	Chinese	Male	54	Squamous carcinoma	ⅢB	Mid-Ad
C1802993	Chinese	Female	60	Squamous carcinoma	ⅢB	Mid-Ad
C1802994	Chinese	Male	57	Squamous carcinoma	ⅢB	Mid-Ad
C1802999	Chinese	Male	66	Squamous carcinoma	ⅢB	Mid-Ad
C1803010	Chinese	Male	53	Squamous carcinoma	ⅢB	Mid-Ad
C1803012	Chinese	Male	68	Squamous carcinoma	ⅢB	Mid-Ad
C1803021	Chinese	Female	64	Squamous carcinoma	ⅢB	Mid-Ad
C1803048	Chinese	Male	57	Squamous carcinoma	ⅢB	Mid-Ad
C1803079	Chinese	Female	50	Squamous carcinoma	ⅢB	Mid-Ad
C1906086	Chinese	Female	59	Squamous carcinoma	ⅢB	Mid-Ad
Z18W01297	Chinese	Male	59	Squamous carcinoma	ⅣA	Mid-Ad
C1803006	Chinese	Male	55	Squamous carcinoma	ⅣA	Mid-Ad
C1803008	Chinese	Male	70	Squamous carcinoma	ⅣA	Mid-Ad
C1803084	Chinese	Female	67	Squamous carcinoma	ⅣA	Mid-Ad
C1906078	Chinese	Female	55	-	-	HC
C1906079	Chinese	Male	56	-	-	HC
C1906080	Chinese	Male	57	-	-	HC
C1803054	Chinese	Male	53	-	-	HC
C1803056	Chinese	Male	48	-	-	HC
C1803055	Chinese	Male	56	-	-	HC
C1803057	Chinese	Male	58	-	-	HC
C1803058	Chinese	Male	63	-	-	HC
C1803059	Chinese	Male	53	-	-	HC
C1803060	Chinese	Female	51	-	-	HC
C1803063	Chinese	Female	60	-	-	HC
C1803064	Chinese	Male	56	-	-	HC
C1803065	Chinese	Female	56	-	-	HC
C1803067	Chinese	Female	58	-	-	HC
C1803066	Chinese	Female	55	-	-	HC
C1906083	Chinese	Male	63	-	-	HC
C1803068	Chinese	Male	69	-	-	HC
C1803073	Chinese	Male	57	-	-	HC
C1803074	Chinese	Male	64	-	-	HC
C2005344	Chinese	Male	67	-	-	HC
C1906088	Chinese	Male	57	-	-	HC

C1906089	Chinese	Female	54	-	-	HC
C1906090	Chinese	Female	67	-	-	HC
C1906091	Chinese	Male	61	-	-	HC
C1906092	Chinese	Male	52	-	-	HC
C1906094	Chinese	Male	52	-	-	HC
C1906097	Chinese	Male	51	-	-	HC
C1906098	Chinese	Male	50	-	-	HC
C2005312	Chinese	Male	52	-	-	HC
C2005313	Chinese	Male	52	-	-	HC
C2005314	Chinese	Female	57	-	-	HC
C2005315	Chinese	Female	52	-	-	HC
C2005316	Chinese	Male	58	-	-	HC
C2005317	Chinese	Male	68	-	-	HC
C2005318	Chinese	Male	58	-	-	HC
C2005321	Chinese	Male	61	-	-	HC
C2005322	Chinese	Male	63	-	-	HC
C2005323	Chinese	Male	58	-	-	HC
C2005324	Chinese	Female	60	-	-	HC
C2005331	Chinese	Male	65	-	-	HC
C2005332	Chinese	Male	70	-	-	HC
C2005333	Chinese	Male	63	-	-	HC
C2005334	Chinese	Male	65	-	-	HC
C2005335	Chinese	Male	61	-	-	HC
C2005336	Chinese	Male	51	-	-	HC
C2005337	Chinese	Female	52	-	-	HC
C2005338	Chinese	Female	66	-	-	HC
C2005339	Chinese	Male	54	-	-	HC
C2005342	Chinese	Male	61	-	-	HC
C2005341	Chinese	Male	69	-	-	HC
C1801631	Chinese	female	50	-	-	HC
C180109Z	Chinese	male	unknown	-	-	HC
C1801479	Chinese	female	43	-	-	HC
C1801501	Chinese	male	44	-	-	HC
C1801200	Chinese	male	67	-	-	HC
C1800147	Chinese	male	56	-	-	HC
C1801406	Chinese	male	53	-	-	HC
C1800170	Chinese	male	49	-	-	HC
C1801303	Chinese	male	54	-	-	HC
C1800129	Chinese	male	43	-	-	HC
C1800116	Chinese	female	40	-	-	HC
C1800117	Chinese	male	45	-	-	HC
C1801453	Chinese	male	68	-	-	HC

C1801224	Chinese	male	60	-	-	HC
C1801418	Chinese	male	70	-	-	HC
C1801554	Chinese	male	59	-	-	HC
C1801565	Chinese	male	69	-	-	HC
C1801656	Chinese	male	71	-	-	HC
C1801233	Chinese	male	65	-	-	HC
C1801516	Chinese	male	67	-	-	HC
C1801524	Chinese	female	49	-	-	HC

Note: Mid-Ad, middle to advanced; HC, healthy controls.

Table S2 List of annotated 5hmC marker genes used in model construction.

seqnames	start	end	width	annotation	geneChr	geneStart
chr19	46436992	46477123	40132	Promoter (<=1kb)	19	46442771
chr19	48216679	48246391	29713	Promoter (<=1kb)	19	48216601
chr19	48741518	48759203	17686	Promoter (<=1kb)	19	48758932
chr19	48829256	48833810	4555	Promoter (<=1kb)	19	48828629
chr19	49223842	49243867	20026	Promoter (<=1kb)	19	49223842
chr19	49458132	49465055	6924	Promoter (<=1kb)	19	49458117
chr19	50433461	50437193	3733	Promoter (<=1kb)	19	50436321
chr19	51279398	51289467	10070	)21uyg.1/uc021uyg.1, ex(	19	51293672
chr19	55141968	55149007	7040	Promoter (<=1kb)	19	55141968
chr19	56511092	56573176	62085	Promoter (<=1kb)	19	56511092
chr19	56613019	56632664	19646	Promoter (<=1kb)	19	56598732
chr2	10508890	10567743	58854	Promoter (<=1kb)	2	10508890
chr2	12147242	12718474	571233	002rbu.1/uc002rbu.1, exo	2	12856998
chr2	16730727	16847102	116376	Promoter (<=1kb)	2	16730730
chr2	27938990	27962154	23165	Distal Intergenic	2	27994584
chr2	27994586	28002600	8015	Promoter (<=1kb)	2	27994584
chr2	38294746	38303323	8578	Promoter (<=1kb)	2	38294746
chr2	44544746	44589001	44256	Promoter (<=1kb)	2	44544748
chr2	46524546	46613836	89291	Promoter (<=1kb)	2	46606839
chr2	48541821	48606434	64614	Promoter (<=1kb)	2	48541795
chr2	54785531	54889445	103915	Promoter (<=1kb)	2	54785531
chr2	61704984	61765491	60508	Promoter (<=1kb)	2	61705069
chr2	62442656	62451866	9211	5' UTR	2	62432961
chr2	64319786	64371554	51769	Promoter (<=1kb)	2	64319786
chr2	65313988	65357435	43448	Promoter (<=1kb)	2	65313988
chr2	65454829	65498387	43559	Promoter (<=1kb)	2	65454829
chr2	66650474	66660602	10129	Promoter (<=1kb)	2	66650475
chr2	68405989	68479664	73676	Promoter (<=1kb)	2	68405989
chr2	74379725	74405441	25717	Promoter (<=1kb)	2	74383211
chr2	74781287	74784678	3392	Promoter (<=1kb)	2	74781512
chr2	85198284	85286595	88312	Promoter (<=1kb)	2	85198231
chr2	95534430	95613087	78658	Promoter (<=1kb)	2	95537232
chr2	99764727	99771429	6703	Promoter (<=1kb)	2	99771418
chr2	1.02E+08	1.02E+08	33101	Promoter (<=1kb)	2	1.02E+08
chr2	1.07E+08	1.07E+08	9282	Promoter (<=1kb)	2	1.07E+08
chr2	1.12E+08	1.12E+08	65853	Promoter (<=1kb)	2	1.12E+08
chr2	1.13E+08	1.13E+08	17887	Promoter (<=1kb)	2	1.13E+08
chr2	1.14E+08	1.14E+08	25435	Promoter (<=1kb)	2	1.14E+08
chr2	1.15E+08	1.15E+08	70957	Promoter (<=1kb)	2	1.15E+08
chr2	1.28E+08	1.28E+08	43365	Promoter (<=1kb)	2	1.28E+08
chr2	1.36E+08	1.36E+08	40521	Promoter (<=1kb)	2	1.36E+08
chr2	1.53E+08	1.54E+08	314653	Promoter (<=1kb)	2	1.53E+08
chr2	1.75E+08	1.75E+08	59244	Promoter (<=1kb)	2	1.75E+08
chr2	1.78E+08	1.78E+08	34420	Promoter (<=1kb)	2	1.78E+08
chr2	1.78E+08	1.79E+08	293438	Promoter (<=1kb)	2	1.78E+08
chr2	1.92E+08	1.92E+08	38714	Promoter (<=1kb)	2	1.92E+08
chr2	2.02E+08	2.02E+08	32414	Promoter (<=1kb)	2	2.02E+08

chr2	2.02E+08	2.02E+08	84859	Promoter (<=1kb)	2	2.02E+08
chr2	2.08E+08	2.08E+08	46867	Promoter (<=1kb)	2	2.08E+08
chr2	2.14E+08	2.14E+08	11895	Distal Intergenic	2	2.14E+08
chr2	2.18E+08	2.18E+08	4870	3' UTR	2	2.17E+08
chr2	2.19E+08	2.19E+08	36952	Promoter (<=1kb)	2	2.19E+08
chr2	2.19E+08	2.19E+08	14622	Promoter (<=1kb)	2	2.19E+08
chr2	2.35E+08	2.35E+08	2973	Promoter (<=1kb)	2	2.35E+08
chr2	2.39E+08	2.39E+08	73711	Promoter (<=1kb)	2	2.39E+08
chr2	2.39E+08	2.39E+08	6565	Promoter (<=1kb)	2	2.39E+08
chr20	627259	633844	6586	Promoter (<=1kb)	20	627268
chr20	825286	826920	1635	Promoter (<=1kb)	20	825285
chr20	3870463	3910534	40072	Promoter (<=1kb)	20	3898149
chr20	5525085	5591652	66568	Promoter (<=1kb)	20	5525080
chr20	10618332	10654647	36316	Promoter (<=1kb)	20	10622108
chr20	19870210	19983103	112894	Promoter (<=1kb)	20	19870210
chr20	23614294	23618592	4299	Promoter (<=1kb)	20	23608534
chr20	35521096	35580111	59016	Promoter (<=1kb)	20	35520227
chr20	36145819	36156333	10515	Promoter (<=1kb)	20	36145819
chr20	36766345	36793672	27328	Promoter (<=1kb)	20	36756864
chr20	37434340	37551667	117328	Promoter (<=1kb)	20	37434348
chr20	42574536	42698256	123721	Promoter (<=1kb)	20	42574536
chr20	44563322	44576659	13338	Promoter (<=1kb)	20	44573507
chr20	45186467	45313124	126658	Promoter (<=1kb)	20	45186462
chr20	46130631	46285616	154986	Promoter (<=1kb)	20	46255746
chr20	48519928	48532066	12139	Promoter (<=1kb)	20	48519929
chr20	49126920	49201778	74859	Promoter (<=1kb)	20	49126891
chr20	55966452	55984389	17938	Promoter (<=1kb)	20	55966454
chr20	57467218	57475554	8337	Promoter (<=1kb)	20	57472495
chr20	60697531	60710430	12900	Promoter (<=1kb)	20	60698193
chr20	60878053	60883918	5866	Promoter (<=1kb)	20	60880362
chr20	61273855	61303645	29791	Promoter (<=1kb)	20	61294379
chr20	62185512	62188048	2537	Promoter (1-2kb)	20	62184373
chr21	30677525	30734217	56693	Promoter (<=1kb)	21	30677560
chr21	34602243	34637969	35727	Promoter (<=1kb)	21	34621014
chr21	34775202	34809828	34627	Promoter (<=1kb)	21	34775202
chr21	45553565	45565593	12029	Promoter (<=1kb)	21	45553494
chr22	17618410	17646177	27768	Promoter (<=1kb)	22	17640279
chr22	20119327	20135530	16204	Promoter (<=1kb)	22	20119364
chr22	21922003	21978323	56321	Promoter (<=1kb)	22	21922019
chr22	22123319	22221970	98652	Promoter (<=1kb)	22	22113947
chr22	31644370	31676066	31697	Promoter (<=1kb)	22	31644348
chr22	36677323	36784112	106790	Promoter (<=1kb)	22	36677323
chr22	43265779	43411155	145377	Promoter (<=1kb)	22	43265772
chr22	45714324	45737836	23513	Promoter (<=1kb)	22	45725525
chr22	46481877	46509808	27932	Promoter (<=1kb)	22	46486924
chr22	47169824	47571342	401519	Promoter (<=1kb)	22	47169824
chr22	50964181	50968514	4334	Promoter (<=1kb)	22	50964181
chr3	9799031	9811631	12601	Promoter (<=1kb)	3	9799031
chr3	14444106	14530857	86752	Promoter (<=1kb)	3	14444076
chr3	18486729	18571606	84878	Distal Intergenic	3	18389133

chr3	30648093	30735634	87542	Promoter (<=1kb)	3	30647994
chr3	32433345	32497020	63676	Promoter (<=1kb)	3	32433163
chr3	38738837	38835501	96665	Promoter (<=1kb)	3	38738837
chr3	41240996	41281934	40939	Promoter (<=1kb)	3	41277163
chr3	46395602	46402420	6819	Promoter (<=1kb)	3	46395235
chr3	49396578	49449409	52832	Promoter (<=1kb)	3	49396579
chr3	50712358	51421629	709272	Promoter (<=1kb)	3	50712511
chr3	52082935	52090587	7653	Promoter (<=1kb)	3	52082937
chr3	52489620	52527084	37465	Promoter (<=1kb)	3	52518408
chr3	57994149	58157978	163830	Promoter (<=1kb)	3	58063983
chr3	60873925	61237126	363202	Promoter (<=1kb)	3	59735036
chr3	1.09E+08	1.09E+08	46052	Promoter (<=1kb)	3	1.09E+08
chr3	1.25E+08	1.25E+08	90288	Promoter (<=1kb)	3	1.25E+08
chr3	1.25E+08	1.25E+08	130130	Promoter (<=1kb)	3	1.25E+08
chr3	1.29E+08	1.29E+08	73662	Promoter (<=1kb)	3	1.29E+08
chr3	1.32E+08	1.33E+08	152462	Promoter (<=1kb)	3	1.32E+08
chr3	1.36E+08	1.36E+08	47419	Promoter (<=1kb)	3	1.36E+08
chr3	1.52E+08	1.52E+08	166376	Promoter (<=1kb)	3	1.52E+08
chr3	1.75E+08	1.76E+08	951213	Promoter (<=1kb)	3	1.75E+08
chr3	1.93E+08	1.93E+08	121042	Promoter (<=1kb)	3	1.93E+08
chr3	1.96E+08	1.96E+08	22311	Promoter (<=1kb)	3	1.96E+08
chr3	1.96E+08	1.97E+08	92791	Promoter (<=1kb)	3	1.96E+08
chr4	8201017	8242830	41814	Promoter (<=1kb)	4	8201060
chr4	26483018	26492106	9089	Promoter (<=1kb)	4	26483018
chr4	38665820	38703138	37319	Promoter (<=1kb)	4	38614322
chr4	56212409	56239267	26859	Promoter (<=1kb)	4	56212388
chr4	56262124	56292342	30219	Promoter (<=1kb)	4	56262080
chr4	71457973	71473005	15033	Promoter (<=1kb)	4	71457975
chr4	1.21E+08	1.21E+08	5410	:003idk.1/uc003idk.1, exo	4	1.2E+08
chr4	1.42E+08	1.42E+08	273663	Promoter (<=1kb)	4	1.42E+08
chr4	1.59E+08	1.59E+08	48552	Promoter (<=1kb)	4	1.59E+08
chr4	1.6E+08	1.6E+08	92365	Promoter (<=1kb)	4	1.6E+08
chr4	1.85E+08	1.85E+08	86822	Promoter (<=1kb)	4	1.85E+08
chr5	9035145	9546187	511043	Promoter (<=1kb)	5	9053928
chr5	14143451	14510313	366863	Promoter (<=1kb)	5	14143829
chr5	43016150	43018913	2764	Promoter (<=1kb)	5	43014831
chr5	53813589	53842416	28828	Promoter (<=1kb)	5	53813589
chr5	55807109	55902083	94975	Distal Intergenic	5	56110900
chr5	56111376	56191979	80604	Promoter (<=1kb)	5	56110900
chr5	67588405	67597649	9245	Promoter (<=1kb)	5	67588511
chr5	73923231	73937249	14019	Promoter (<=1kb)	5	73935848
chr5	81569139	81574173	5035	Promoter (<=1kb)	5	81569139
chr5	1.02E+08	1.02E+08	62973	:003knr.1/uc003knr.1, exo	5	1.02E+08
chr5	1.09E+08	1.09E+08	75253	Promoter (<=1kb)	5	1.09E+08
chr5	1.28E+08	1.28E+08	68148	Promoter (<=1kb)	5	1.28E+08
chr5	1.32E+08	1.32E+08	65116	Promoter (<=1kb)	5	1.32E+08
chr5	1.38E+08	1.38E+08	59635	Promoter (<=1kb)	5	1.38E+08
chr5	1.39E+08	1.39E+08	37928	Promoter (<=1kb)	5	1.39E+08
chr5	1.39E+08	1.39E+08	67096	Promoter (<=1kb)	5	1.39E+08
chr5	1.4E+08	1.4E+08	58067	Promoter (<=1kb)	5	1.4E+08

chr5	1.41E+08	1.41E+08	15759	Promoter (<=1kb)	5	1.41E+08
chr5	1.42E+08	1.43E+08	457785	Promoter (<=1kb)	5	1.42E+08
chr5	1.48E+08	1.48E+08	172976	Promoter (<=1kb)	5	1.48E+08
chr5	1.49E+08	1.49E+08	58458	Promoter (<=1kb)	5	1.49E+08
chr5	1.5E+08	1.5E+08	10477	Distal Intergenic	5	1.5E+08
chr5	1.5E+08	1.5E+08	8398	Promoter (<=1kb)	5	1.5E+08
chr5	1.71E+08	1.72E+08	146146	Promoter (<=1kb)	5	1.71E+08
chr5	1.77E+08	1.77E+08	5385	Promoter (<=1kb)	5	1.77E+08
chr5	1.79E+08	1.79E+08	17174	Promoter (<=1kb)	5	1.79E+08
chr5	1.8E+08	1.8E+08	2448	)11dhb.1/uc011dhb.1, ex	5	1.8E+08
chr5	1.8E+08	1.8E+08	20736	Promoter (<=1kb)	5	1.8E+08
chr6	12290594	12297427	6834	Promoter (<=1kb)	6	12290529
chr6	16129317	16148479	19163	Promoter (<=1kb)	6	16141787
chr6	18522978	18723129	200152	Promoter (<=1kb)	6	18572015
chr6	36646491	36655109	8619	Promoter (<=1kb)	6	36646487
chr6	39657754	39693199	35446	Promoter (<=1kb)	6	39302876
chr6	40846625	40991019	144395	Distal Intergenic	6	40994640
chr6	44081191	44095228	14038	Promoter (<=1kb)	6	44081373
chr6	46820259	46922676	102418	Promoter (<=1kb)	6	46820242
chr6	49801970	49844809	42840	Promoter (<=1kb)	6	49801979
chr6	52362206	52441858	79653	Promoter (<=1kb)	6	52370340
chr6	52761444	52774496	13053	Promoter (<=1kb)	6	52761439
chr6	53512714	53530554	17841	Promoter (<=1kb)	6	53512699
chr6	62389866	62996130	606265	Promoter (<=1kb)	6	62389865
chr6	88384578	88411951	27374	Promoter (<=1kb)	6	88384578
chr6	1.01E+08	1.01E+08	74802	Promoter (<=1kb)	6	1.01E+08
chr6	1.1E+08	1.1E+08	21907	Promoter (<=1kb)	6	1.1E+08
chr6	1.11E+08	1.12E+08	143692	Promoter (<=1kb)	6	1.11E+08
chr6	1.33E+08	1.33E+08	51643	Promoter (<=1kb)	6	1.33E+08
chr6	1.37E+08	1.37E+08	32989	Promoter (<=1kb)	6	1.37E+08
chr6	1.39E+08	1.4E+08	45723	Promoter (<=1kb)	6	1.39E+08
chr6	1.44E+08	1.44E+08	73870	Promoter (<=1kb)	6	1.44E+08
chr6	1.45E+08	1.45E+08	561298	Promoter (<=1kb)	6	1.45E+08
chr6	1.49E+08	1.49E+08	42900	Promoter (<=1kb)	6	1.49E+08
chr6	1.58E+08	1.58E+08	121832	Promoter (<=1kb)	6	1.58E+08
chr6	1.59E+08	1.59E+08	99000	Promoter (<=1kb)	6	1.59E+08
chr6	1.59E+08	1.59E+08	8270	Promoter (<=1kb)	6	1.59E+08
chr6	1.59E+08	1.59E+08	16516	Promoter (<=1kb)	6	1.59E+08
chr6	1.6E+08	1.6E+08	2410	Promoter (<=1kb)	6	1.6E+08
chr6	1.64E+08	1.64E+08	163875	Promoter (<=1kb)	6	1.64E+08
chr7	881011	914571	33561	Promoter (<=1kb)	7	882978
chr7	2559408	2568811	9404	Promoter (<=1kb)	7	2566708
chr7	4721926	4811073	89148	Promoter (<=1kb)	7	4721930
chr7	5553485	5565176	11692	Promoter (<=1kb)	7	5528809
chr7	5632447	5646286	13840	Promoter (<=1kb)	7	5642503
chr7	5659678	5821294	161617	Promoter (<=1kb)	7	5702063
chr7	6201410	6312255	110846	Promoter (<=1kb)	7	6201412
chr7	6414158	6443598	29441	Promoter (<=1kb)	7	6414126
chr7	6448760	6487585	38826	Promoter (<=1kb)	7	6448747
chr7	26191818	26226757	34940	Promoter (<=1kb)	7	26191847

chr7	30464143	30518482	54340	Promoter (<=1kb)	7	30464143
chr7	31377079	31380340	3262	Promoter (<=1kb)	7	31377075
chr7	32907784	32931372	23589	Promoter (<=1kb)	7	32907778
chr7	36892511	37025871	133361	Promoter (<=1kb)	7	36958962
chr7	43152246	43605600	453355	Promoter (<=1kb)	7	43190494
chr7	44111661	44122139	10479	Promoter (<=1kb)	7	44111847
chr7	48128862	48148333	19472	Promoter (<=1kb)	7	48128851
chr7	55538306	55640195	101890	Promoter (<=1kb)	7	55538306
chr7	71244476	71912128	667653	Promoter (<=1kb)	7	71244476
chr7	73588686	73611426	22741	Promoter (<=1kb)	7	73605528
chr7	74072044	74175022	102979	Promoter (<=1kb)	7	74163362
chr7	75162619	75368259	205641	Promoter (<=1kb)	7	75162619
chr7	75956116	75988308	32193	Promoter (<=1kb)	7	75956108
chr7	93053800	93204042	150243	Promoter (<=1kb)	7	93113248
chr7	98625063	98741731	116669	Promoter (<=1kb)	7	98625058
chr7	98972353	98992439	20087	Promoter (<=1kb)	7	98972298
chr7	1E+08	1E+08	4288	Promoter (<=1kb)	7	1E+08
chr7	1E+08	1E+08	12696	Promoter (<=1kb)	7	1E+08
chr7	1.02E+08	1.02E+08	29423	Promoter (<=1kb)	7	1.02E+08
chr7	1.03E+08	1.03E+08	71971	Promoter (<=1kb)	7	1.03E+08
chr7	1.04E+08	1.05E+08	579902	Promoter (<=1kb)	7	1.04E+08
chr7	1.05E+08	1.05E+08	9419	Promoter (<=1kb)	7	1.05E+08
chr7	1.05E+08	1.05E+08	100815	Promoter (<=1kb)	7	1.05E+08
chr7	1.07E+08	1.07E+08	37755	Promoter (<=1kb)	7	1.07E+08
chr7	1.16E+08	1.16E+08	34818	Promoter (<=1kb)	7	1.16E+08
chr7	1.17E+08	1.17E+08	59463	Promoter (<=1kb)	7	1.17E+08
chr7	1.24E+08	1.24E+08	13519	Promoter (<=1kb)	7	1.24E+08
chr7	1.31E+08	1.31E+08	165757	Promoter (<=1kb)	7	1.31E+08
chr7	1.31E+08	1.31E+08	56358	Promoter (<=1kb)	7	1.31E+08
chr7	1.35E+08	1.35E+08	5047	Promoter (<=1kb)	7	1.35E+08
chr7	1.39E+08	1.39E+08	49381	Promoter (<=1kb)	7	1.39E+08
chr7	1.43E+08	1.43E+08	7663	Promoter (<=1kb)	7	1.43E+08
chr7	1.5E+08	1.5E+08	28764	Promoter (<=1kb)	7	1.5E+08
chr7	1.5E+08	1.5E+08	7274	Promoter (<=1kb)	7	1.5E+08
chr8	1711955	1734736	22782	Promoter (<=1kb)	8	1711870
chr8	8175258	8243949	68692	Promoter (<=1kb)	8	8175258
chr8	11700033	11725596	25564	Promoter (<=1kb)	8	11700034
chr8	21766384	21771201	4818	Promoter (<=1kb)	8	21563902
chr8	23082734	23088439	5706	Promoter (<=1kb)	8	23082734
chr8	24153327	24406131	252805	Promoter (<=1kb)	8	24184067
chr8	26150732	26230196	79465	Promoter (<=1kb)	8	26150732
chr8	26435921	26515691	79771	Promoter (<=1kb)	8	26435921
chr8	28203107	28243977	40871	Promoter (<=1kb)	8	28203102
chr8	28924795	29120587	195793	Promoter (<=1kb)	8	28924795
chr8	29779029	29811125	32097	Promoter (<=1kb)	8	29779029
chr8	29952924	29995864	42941	Promoter (<=1kb)	8	29952922
chr8	40388109	40755345	367237	Promoter (<=1kb)	8	40388111
chr8	41799567	41909505	109939	Promoter (<=1kb)	8	41786997
chr8	42032236	42065083	32848	Promoter (<=1kb)	8	42032236
chr8	42128820	42189978	61159	Promoter (<=1kb)	8	42128820

chr8	56792394	56926728	134335	Promoter (<=1kb)	8	56792386
chr8	61429546	61536203	106658	Promoter (<=1kb)	8	61429469
chr8	91804003	91971621	167619	Promoter (<=1kb)	8	91952967
chr8	96281064	96822371	541308	Promoter (<=1kb)	8	96281064
chr8	97274130	97349223	75094	Promoter (<=1kb)	8	97274167
chr8	1.01E+08	1.01E+08	145114	Promoter (<=1kb)	8	1.01E+08
chr8	1.29E+08	1.29E+08	306721	Promoter (<=1kb)	8	1.29E+08
chr8	1.31E+08	1.31E+08	391571	Promoter (<=1kb)	8	1.31E+08
chr8	1.34E+08	1.34E+08	60078	Promoter (<=1kb)	8	1.34E+08
chr8	1.34E+08	1.35E+08	117040	Promoter (<=1kb)	8	1.34E+08
chr8	1.39E+08	1.39E+08	273872	)22bbu.1/uc022bbu.1, ex	8	1.39E+08
chr8	1.42E+08	1.42E+08	115478	Promoter (<=1kb)	8	1.42E+08
chr8	1.42E+08	1.42E+08	67216	Promoter (<=1kb)	8	1.42E+08
chr8	1.42E+08	1.42E+08	16647	Promoter (<=1kb)	8	1.42E+08
chr9	5163868	5185639	21772	Promoter (<=1kb)	9	5163863
chr9	8314246	10613002	2298757	Promoter (<=1kb)	9	8314246
chr9	19507450	19787017	279568	Promoter (<=1kb)	9	19507450
chr9	33817158	33920397	103240	Promoter (<=1kb)	9	33817182
chr9	35056061	35072665	16605	Promoter (<=1kb)	9	35057373
chr9	37668006	37746901	78896	Promoter (<=1kb)	9	37715635
chr9	72435733	72521143	85411	Promoter (<=1kb)	9	72435731
chr9	73398773	74061887	663115	Promoter (<=1kb)	9	73424891
chr9	74966341	74979508	13168	Promoter (<=1kb)	9	74966341
chr9	80331013	80646727	315715	Promoter (<=1kb)	9	80335191
chr9	95858499	95875565	17067	Promoter (<=1kb)	9	95858450
chr9	99837953	99844227	6275	Promoter (<=1kb)	9	99837953

Table S3A. List of 5' end motif biomarkers used in model construction.

Motif
AAAT
AGTC
AAAA
AAAG
AGTT
GTAG
TTAC
TTAA
GAAA
GAAG
GCTA
ATAA
CCAC
ATAT
CCAG
TAAA
TGTA
CTCT
CTCC
TCTA
CTCG
CTAG
AAGC
ACGT
AAGA
GTGA
GTGG
CTAT
GGCA
GTGT
TTCT
AGGA
AGGC
GATT
GACT
AGGT
TCAC

TTCA
TTCC
GACC
GATA
GATC
GCCC
GGAC
CCTT
GCCT
ATCC
ATCA
CCTC
CCTG
CAGG
TGGG
TGGC
TGGA
TCGA
TGGT
TAGG
ACTG
TCGT
TAGA
ACAG
ACAC
ACAA
AGAC
AGAA
ATTT
AGAG
GGAG
ATTG
ATTC
ATTA
TGAT
TTTA
TTTC
CAAC
GCAC

CTTC
AATT
TATG
TCAA
AATA
AATG
CTGC
TATT
GTTC
GTCA
AACA
CTTA
ACCC
GGGC
CTTT
ACCT
TGTT
AACT
GAGT
AGCA
TTGT
GAGA
TTGA
TTGC
TTGG
CACT
ATGA
ATGG
GGTC
GCGC
GCAA
TGCC
CCCA
TGCA
CACA
CAAA
CACC
TACA
TACC

CATA
CATG
TCCT
TCCC
TCCA

Table S3B. List of NF biomarkers used in model construction.

Chr	Region	Start	End	Strand	Gene name	Transcript id
Chr1	protein_coding	1321862	1333684	-	CCNL2	ENST00000408952
Chr1	protein_coding	3.7E+07	3.7E+07	-	EVA1B	ENST00000270824
Chr1	protein_coding	4.1E+07	4.1E+07	+	CTPS1	ENST00000372616
Chr1	protein_coding	4.4E+07	4.4E+07	+	ST3GAL3	ENST00000372369
Chr1	protein_coding	4.5E+07	4.5E+07	+	BTBD19	ENST00000450269
Chr1	protein_coding	6.8E+07	6.8E+07	+	IL23R	ENST00000395227
Chr1	protein_coding	1E+08	1E+08	+	AMY2A	ENST00000414303
Chr1	protein_coding	1.5E+08	1.5E+08	+	CRCT1	ENST00000368790
Chr1	protein_coding	1.6E+08	1.6E+08	+	OR10Z1	ENST00000361284
Chr1	lincRNA	1.8E+08	1.8E+08	-	GS1-122H1.2	ENST00000608183
Chr1	protein_coding	2.1E+08	2.1E+08	-	CD34	ENST00000356522
Chr1	protein_coding	2.3E+08	2.3E+08	-	HIST3H3	ENST00000366696
Chr1	protein_coding	2.4E+08	2.4E+08	+	FMN2	ENST00000447095
Chr2	protein_coding	3.7E+07	3.7E+07	-	AC007382.1	ENST00000593798
Chr2	protein_coding	4.5E+07	4.5E+07	+	CAMKMT	ENST00000428929
Chr2	protein_coding	4.7E+07	4.7E+07	-	CALM2	ENST00000272298
Chr2	protein_coding	1E+08	1E+08	+	PDCL3	ENST00000416255
Chr2	lincRNA	1.2E+08	1.2E+08	-	AC093901.1	ENST00000414886
Chr2	protein_coding	1.6E+08	1.6E+08	+	GALNT13	ENST00000422126
Chr2	protein_coding	1.7E+08	1.7E+08	-	GRB14	ENST00000263915
Chr2	protein_coding	1.7E+08	1.7E+08	+	B3GALT1	ENST00000392690
Chr2	protein_coding	2E+08	2E+08	-	STK17B	ENST00000449152
Chr2	protein_coding	2E+08	2E+08	-	HSPD1	ENST00000426480
Chr2	protein_coding	2E+08	2E+08	+	NOP58	ENST00000264279
Chr2	protein_coding	2.2E+08	2.2E+08	-	MREG	ENST00000424992
Chr2	protein_coding	2.3E+08	2.3E+08	-	GPR55	ENST00000392039
Chr2	protein_coding	2.3E+08	2.3E+08	+	C2orf72	ENST00000373640
Chr2	protein_coding	2.3E+08	2.3E+08	+	INPP5D	ENST00000415617
Chr2	protein_coding	2.3E+08	2.3E+08	+	UGT1A8	ENST00000609767
Chr2	protein_coding	2.3E+08	2.3E+08	+	UGT1A3	ENST00000482026
Chr2	protein_coding	2.4E+08	2.4E+08	-	KIF1A	ENST00000498729
Chr3	protein_coding	9851672	9877173	+	TTLL3	ENST00000426895
Chr3	protein_coding	4E+07	4E+07	+	MOBP	ENST00000451925
Chr3	protein_coding	5E+07	5E+07	-	TMEM115	ENST00000266025
Chr3	protein_coding	5.2E+07	5.2E+07	+	ALAS1	ENST00000394965
Chr3	protein_coding	1.1E+08	1.1E+08	+	GTPBP8	ENST00000383677
Chr3	protein_coding	1.3E+08	1.3E+08	+	CEP63	ENST00000514678
Chr3	lincRNA	2E+08	2E+08	+	LINC00969	ENST00000596584
Chr3	protein_coding	2E+08	2E+08	-	KIAA0226	ENST00000273582

Chr4	protein_coding	4269428	4291896	-	LYAR	ENST00000343470
Chr4	protein_coding	4291924	4323512	+	ZBTB49	ENST00000355834
Chr4	lincRNA	1.8E+08	1.8E+08	-	RP11-440I14.2	ENST00000515178
Chr4	protein_coding	1.8E+08	1.8E+08	-	ASB5	ENST00000505299
Chr5	lincRNA	1363697	1380182	-	RP11-325I22.2	ENST00000504989
Chr5	lincRNA	2.7E+07	2.7E+07	-	CTD-2533K21.4	ENST00000506032
Chr5	miRNA	2.8E+07	2.8E+07	-	AC010455.1	ENST00000408794
Chr5	protein_coding	3.4E+07	3.4E+07	-	C1QTNF3	ENST00000231338
Chr5	protein_coding	5.5E+07	5.5E+07	-	IL6ST	ENST00000381287
Chr5	protein_coding	6E+07	6E+07	+	NDUFAF2	ENST00000296597
Chr5	protein_coding	6.5E+07	6.5E+07	+	PPWD1	ENST00000505380
Chr5	protein_coding	8.8E+07	8.8E+07	-	MEF2C	ENST00000437473
Chr5	lincRNA	9.1E+07	9.1E+07	-	LUCAT1	ENST00000511918
Chr5	protein_coding	1E+08	1E+08	+	PAM	ENST00000438793
Chr5	protein_coding	1.1E+08	1.1E+08	+	MAN2A1	ENST00000261483
Chr5	protein_coding	1.4E+08	1.4E+08	+	IGIP	ENST00000333305
Chr5	protein_coding	1.6E+08	1.6E+08	+	GABRA1	ENST00000393943
Chr5	protein_coding	1.7E+08	1.7E+08	-	BOD1	ENST00000477985
Chr5	protein_coding	1.8E+08	1.8E+08	-	DOK3	ENST00000357198
Chr5	protein_coding	1.8E+08	1.8E+08	-	CBY3	ENST00000376974
Chr5	protein_coding	1.8E+08	1.8E+08	-	C5orf45	ENST00000403396
Chr6	protein_coding	2.7E+07	2.7E+07	+	ZNF391	ENST00000461521
Chr6	protein_coding	3.2E+07	3.2E+07	+	LST1	ENST00000396101
Chr6	protein_coding	3.3E+07	3.3E+07	-	HLA-DQB2	ENST00000437316
Chr6	protein_coding	7.7E+07	7.7E+07	-	IMPG1	ENST00000369952
Chr6	lincRNA	9.5E+07	9.5E+07	+	RP3-463P15.1	ENST00000424506
Chr6	lincRNA	1.1E+08	1.1E+08	-	RP1-60O19.1	ENST00000602621
Chr7	protein_coding	7273889	7288282	+	C1GALT1	ENST00000223122
Chr7	lincRNA	1.5E+07	1.5E+07	+	AC006458.3	ENST00000445093
Chr7	protein_coding	5E+07	5E+07	+	IKZF1	ENST00000426121
Chr7	protein_coding	7.5E+07	7.5E+07	-	CCL24	ENST00000222902
Chr7	protein_coding	8E+07	8E+07	+	CD36	ENST00000435819
Chr7	protein_coding	9.4E+07	9.4E+07	-	BET1	ENST00000222547
Chr7	miRNA	9.8E+07	9.8E+07	-	MIR5692C2	ENST00000577959
Chr7	protein_coding	1E+08	1E+08	+	PILRB	ENST00000455145
Chr7	protein_coding	1.1E+08	1.1E+08	+	CDHR3	ENST00000542731
Chr7	lincRNA	1.1E+08	1.1E+08	+	AC068610.3	ENST00000460471
Chr7	lincRNA	1.4E+08	1.4E+08	-	RP5-842K16.1	ENST00000461145
Chr7	protein_coding	1.4E+08	1.4E+08	-	TAS2R38	ENST00000547270
Chr7	protein_coding	1.6E+08	1.6E+08	-	BLACE	ENST00000378120
ChrX	protein_coding	2.4E+07	2.4E+07	-	ACOT9	ENST00000379295
ChrX	protein_coding	1.5E+08	1.5E+08	-	CD99L2	ENST00000418547

ChrX	protein_coding	1.5E+08	1.5E+08	-	G6PD	ENST00000369620
Chr8	protein_coding	2.2E+07	2.2E+07	+	CCAR2	ENST00000520738
Chr8	protein_coding	5.4E+07	5.4E+07	-	RB1CC1	ENST00000025008
Chr8	protein_coding	5.5E+07	5.5E+07	-	TCEA1	ENST00000521604
Chr8	lincRNA	7.4E+07	7.4E+07	-	RP11-434I12.4	ENST00000522560
Chr8	protein_coding	8.6E+07	8.6E+07	-	CA1	ENST00000432364
Chr8	protein_coding	9.6E+07	9.6E+07	-	C8orf37	ENST00000286688
Chr8	protein_coding	1.5E+08	1.5E+08	+	MAF1	ENST00000534811
Chr9	lincRNA	1.1E+08	1.1E+08	-	RP11-339N8.1	ENST00000415465
Chr9	protein_coding	1.2E+08	1.2E+08	-	AKNA	ENST00000374075
Chr9	protein_coding	1.3E+08	1.3E+08	+	FPGS	ENST00000373245
Chr9	protein_coding	1.4E+08	1.4E+08	+	C9orf169	ENST00000409414
Chr10	miRNA	2E+07	2E+07	-	AL353147.1	ENST00000390783
Chr10	lincRNA	6.1E+07	6.1E+07	-	LINC00948	ENST00000600486
Chr10	lincRNA	1.1E+08	1.1E+08	-	RP11-215N21.1	ENST00000598903
Chr10	miRNA	1.3E+08	1.3E+08	+	AL360176.1	ENST00000401153
Chr10	miRNA	1.3E+08	1.3E+08	-	AL583860.1	ENST00000408790
Chr10	protein_coding	1.4E+08	1.4E+08	+	CYP2E1	ENST00000252945
Chr11	protein_coding	835194	837513	+	CD151	ENST00000530320
Chr11	protein_coding	5289584	5291388	-	HBE1	ENST00000292896
Chr11	protein_coding	1.1E+07	1.1E+07	-	MRVI1	ENST00000545852
Chr11	protein_coding	3.3E+07	3.3E+07	-	CCDC73	ENST00000528333
Chr11	protein_coding	3.4E+07	3.4E+07	-	CD59	ENST00000415002
Chr11	lincRNA	4.6E+07	4.6E+07	+	CTD-2210P24.1	ENST00000529769
Chr11	protein_coding	6E+07	6E+07	+	STX3	ENST00000529177
Chr11	protein_coding	6E+07	6E+07	+	MS4A3	ENST00000534744
Chr11	protein_coding	6.2E+07	6.2E+07	+	AP003733.1	ENST00000601917
Chr11	protein_coding	7.3E+07	7.3E+07	-	RAB6A	ENST00000310653
Chr11	protein_coding	7.9E+07	7.9E+07	-	TENM4	ENST00000533074
Chr11	miRNA	9.4E+07	9.4E+07	-	MIR548L	ENST00000408303
Chr11	protein_coding	1.1E+08	1.1E+08	+	AP002884.2	ENST00000595053
Chr12	protein_coding	1.9E+07	2E+07	+	PLEKHA5	ENST00000536974
Chr12	protein_coding	5.8E+07	5.8E+07	-	DCTN2	ENST00000546758
Chr12	protein_coding	7.1E+07	7.1E+07	+	CNOT2	ENST00000548159
Chr12	protein_coding	1.2E+08	1.2E+08	+	KNTC1	ENST00000534995
Chr13	protein_coding	2.5E+07	2.5E+07	+	SPATA13	ENST00000424834
Chr13	lincRNA	6.4E+07	6.4E+07	+	RP11-473M10.3	ENST00000418943
Chr13	protein_coding	7.8E+07	7.8E+07	+	IRG1	ENST00000449753
Chr13	lincRNA	1E+08	1E+08	+	RP11-123H22.1	ENST00000444795
Chr14	protein_coding	3.2E+07	3.2E+07	-	HECTD1	ENST00000553700
Chr14	protein_coding	3.2E+07	3.2E+07	-	HEATR5A	ENST00000538864
Chr14	lincRNA	3.6E+07	3.6E+07	-	RP11-561B11.6	ENST00000556448

Chr14	lincRNA	9.9E+07	9.9E+07	+	?P11-1082A3.1	ENST00000554515
Chr15	protein_coding	3.9E+07	3.9E+07	+	SPRED1	ENST00000561317
Chr15	protein_coding	4.3E+07	4.3E+07	+	GANC	ENST00000562859
Chr15	protein_coding	5.4E+07	5.4E+07	-	WDR72	ENST00000559418
Chr15	protein_coding	6.7E+07	6.7E+07	+	DIS3L	ENST00000319194
Chr15	lincRNA	9.8E+07	9.8E+07	+	CTD-2147F2.1	ENST00000560314
Chr16	protein_coding	3701838	3708096	+	DNASE1	ENST00000246949
Chr16	ense_overlappin	3700637	3701704	-	RP11-461A8.4	ENST00000570409
Chr16	protein_coding	4404545	4465898	-	CORO7	ENST00000539968
Chr16	lincRNA	1.8E+07	1.8E+07	-	CTA-481E9.4	ENST00000569048
Chr16	protein_coding	2.1E+07	2.1E+07	+	ACSM3	ENST00000440284
Chr16	protein_coding	3.1E+07	3.1E+07	-	BCL7C	ENST00000215115
Chr16	protein_coding	3.1E+07	3.1E+07	+	ITGAM	ENST00000544665
Chr16	protein_coding	6.9E+07	6.9E+07	+	HAS3	ENST00000219322
Chr16	lincRNA	8.6E+07	8.6E+07	-	RP11-805I24.4	ENST00000600234
Chr17	protein_coding	1.7E+07	1.7E+07	-	PEMT	ENST00000395781
Chr17	protein_coding	3.9E+07	3.9E+07	-	KRTAP1-4	ENST00000377747
Chr17	protein_coding	4.3E+07	4.3E+07	-	GFAP	ENST00000587997
Chr17	protein_coding	6.3E+07	6.3E+07	+	CEP95	ENST00000553412
Chr17	protein_coding	6.3E+07	6.3E+07	-	LRRC37A3	ENST00000334962
Chr18	protein_coding	3455412	3458409	+	TGIF1	ENST00000472042
Chr18	protein_coding	4.6E+07	4.6E+07	+	CTIF	ENST00000587752
Chr18	protein_coding	5.5E+07	5.5E+07	+	ST8SIA3	ENST00000586360
Chr18	protein_coding	6.7E+07	6.8E+07	+	DOK6	ENST00000382713
Chr20	protein_coding	327426	334137	+	NRSN2	ENST00000609179
Chr20	protein_coding	1.8E+07	1.8E+07	-	RRBP1	ENST00000246043
Chr20	protein_coding	3.6E+07	3.6E+07	-	RBL1	ENST00000373664
Chr19	protein_coding	7793843	7797057	-	CLEC4G	ENST00000328853
Chr19	protein_coding	1.2E+07	1.2E+07	-	ZNF44	ENST00000356109
Chr19	protein_coding	1.8E+07	1.8E+07	+	FCHO1	ENST00000596951
Chr19	lincRNA	3.4E+07	3.4E+07	-	;TD-2540B15.1	ENST00000604605
Chr19	protein_coding	3.5E+07	3.5E+07	+	GPI	ENST00000586425
Chr19	protein_coding	3.6E+07	3.6E+07	+	CD22	ENST00000341773
Chr19	protein_coding	3.6E+07	3.6E+07	-	HSPB6	ENST00000592984
Chr19	protein_coding	4.2E+07	4.2E+07	-	ATP5SL	ENST00000586786
Chr19	protein_coding	4.8E+07	4.9E+07	+	ELSPBP1	ENST00000339841
Chr19	protein_coding	4.9E+07	4.9E+07	+	EMP3	ENST00000597279
Chr19	miRNA	5.4E+07	5.4E+07	+	MIR373	ENST00000362273
Chr19	lincRNA	5.4E+07	5.4E+07	+	AC008753.4	ENST00000597420
Chr19	protein_coding	5.4E+07	5.4E+07	+	MYADM	ENST00000336967
Chr19	sense_intronic	5.6E+07	5.6E+07	+	;TD-2611O12.7	ENST00000597680
Chr21	protein_coding	2.3E+07	2.3E+07	+	NCAM2	ENST00000535285

Chr21	lincRNA	2.6E+07	2.6E+07	+	AP000476.1	ENST00000415182
Chr21	protein_coding	3.2E+07	3.2E+07	+	KRTAP20-2	ENST00000330798
Chr21	protein_coding	4.3E+07	4.3E+07	+	MX2	ENST00000418103
Chr21	protein_coding	4.6E+07	4.6E+07	-	UBE2G2	ENST00000330942
Chr21	protein_coding	4.7E+07	4.7E+07	-	PRED57	ENST00000600921

Table S3C. List of fragmentation biomarkers used in model construction.

seqnames	start	end	width	annotation	geneChr	geneStart
chr8	43000002	44000001	1000000	Promoter (<=1kb)	8	43147585
chr4	191000002	192000001	1000000	Promoter (<=1kb)	4	191012063
chr4	134000002	135000001	1000000	Promoter (<=1kb)	4	134070470
chr9	141000002	142000001	1000000	Promoter (<=1kb)	9	141106637
chr1	121000002	122000001	1000000	Promoter (<=1kb)	1	121107152
chr11	69000002	70000001	1000000	Promoter (<=1kb)	11	69471368
chr10	39000002	40000001	1000000	Distal Intergenic	10	38989727
chr14	18000002	19000001	1000000	Distal Intergenic	14	19377594
chr15	19000002	20000001	1000000	Distal Intergenic	15	20487997
chr2	89000002	90000001	1000000	Promoter (<=1kb)	2	89111884

geneEnd	geneLength	geneStrand	distanceToTSS	SYMBOL
43218328	70744	1	0	POTEA
191013442	1380	1	0	DUX4L8
134074404	3935	1	0	PCDH10
141134172	27536	1	0	FAM157B
121129827	22676	1	0	SRGAP2D
69490165	18798	2	0	LTO1
38991371	1645	1	10275	ACTR3BP5
19378574	981	1	-377593	OR11H12
20496811	8815	1	-487996	CHEK2P2
89111968	85	1	0	MIR4436A

GENENAME
POTE ankyrin domain family member A
double homeobox 4 like 8 (pseudogene)
protocadherin 10
family with sequence similarity 157 member B
SLIT-ROBO Rho GTPase activating protein 2D (pseudogene)
LTO1 maturation factor of ABCE1
ACTR3B pseudogene 5
olfactory receptor family 11 subfamily H member 12
checkpoint kinase 2 pseudogene 2
microRNA 4436a

Supplementary Figure Legends

Figure S1. Comparison of subjects' age distributions (A) and gender distributions (B) among HC, Early and Mid-Ad ESCC groups. HC, healthy controls; ESCC, esophageal squamous cell carcinoma; Mid-Ad, middle to advanced.

Figure S2. Study design and research pipeline for early detection of ESCC. 5hmC and low-pass WGS-based diagnostic model were respectively developed for identifying ctDNA from plasma cfDNA using machine learning approach. 171 subjects were analyzed to derive genome-wide 5hmC profiles and further trained and evaluated 5hmC-based diagnostic performance. Due to inadequate cfDNA limitation, only 164 subjects were available to perform low-pass WGS and construct integrated diagnostic model. ctDNA, circulating tumor DNA. cfDNA, cell-free DNA; HC, healthy controls individuals; ESCC, esophageal squamous cell carcinoma; Mid-Ad, middle-advanced; 5hmC, 5-hydroxymethylcytosines; WGS, whole genome sequencing; NF, nucleosome footprint; RFECV, recursive feature elimination - cross validation; Lasso, least absolute shrinkage and selection operator; SVM, support vector machine.

Figure S3. Comparison of the total number of 5hmC peaks between ESCC (green, n=100) and HC (blue, n=71) cohorts. Each dot depicts an individual cfDNA sample.

P value shows statistical significance by Mann-Whitney U test. HC, healthy controls; ESCC, esophageal squamous cell carcinoma.

Figure S4. Comparison of predictive scores of 5hmC classifier among HC, Early ESCC and Mid-Ad ESCC groups in the internal test set. HC, healthy controls; ESCC, esophageal squamous cell carcinoma; Mid-Ad, middle to advanced.

Figure S5. The diagnostic powers of individual genomic features and 5hmC classifier and combined classifier in the test set. (A) The table of diagnostic sensitivity and specificity of individual genomic features in ESCC vs HC of the test set. (B) The table of the diagnostic accuracy of 5hmC classifier and combined classifier in different clinical stages of ESCC. Sen, sensitivity; Spe, specificity; HC, healthy controls; ESCC, esophageal squamous cell carcinoma; NF, nucleosome footprint.

Supplementary Table legends

Table S1. Demographic and clinicopathological characteristics of esophageal cancer patients and healthy controls.

Table S2. List of annotated 5hmC marker genes used in model construction.

Table S3. List of 5’ end motif (A), NF (B) and fragmentation (C) biomarkers used in model construction. NF, nucleosome footprint.

Figure S1

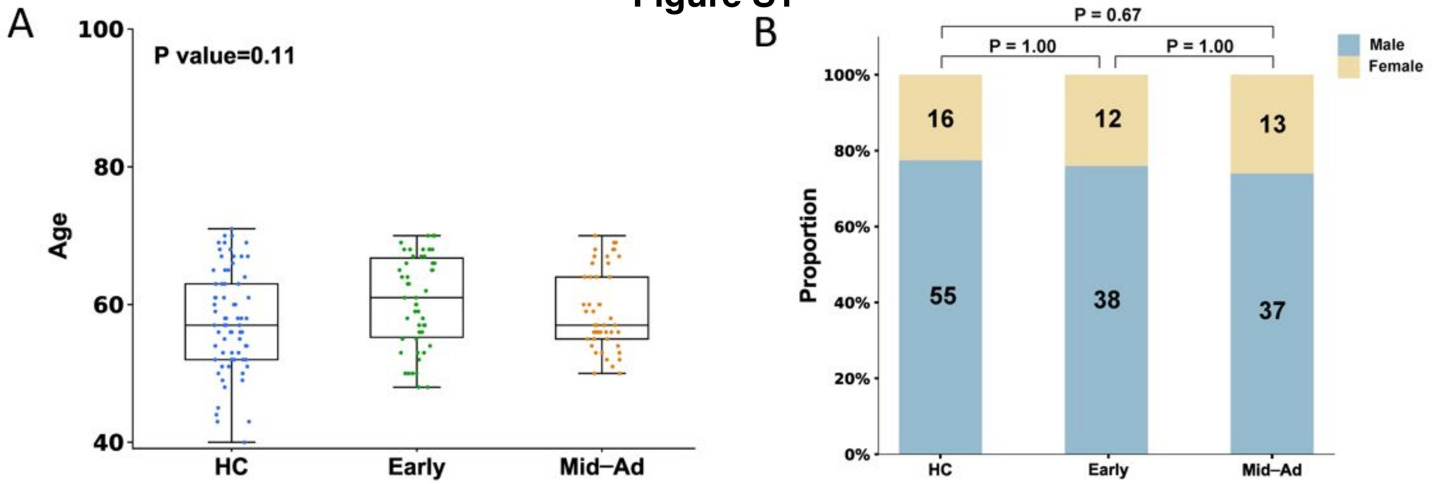

A
P value=0.11
Age
100
80
60
40
HC
Early
Mid−Ad
B
P = 0.67
P = 1.00
P = 1.00
Male
Female
Proportion
100%
80%
60%
40%
20%
0%
16
12
13
55
38
37
HC
Early
Mid−Ad

Figure S2

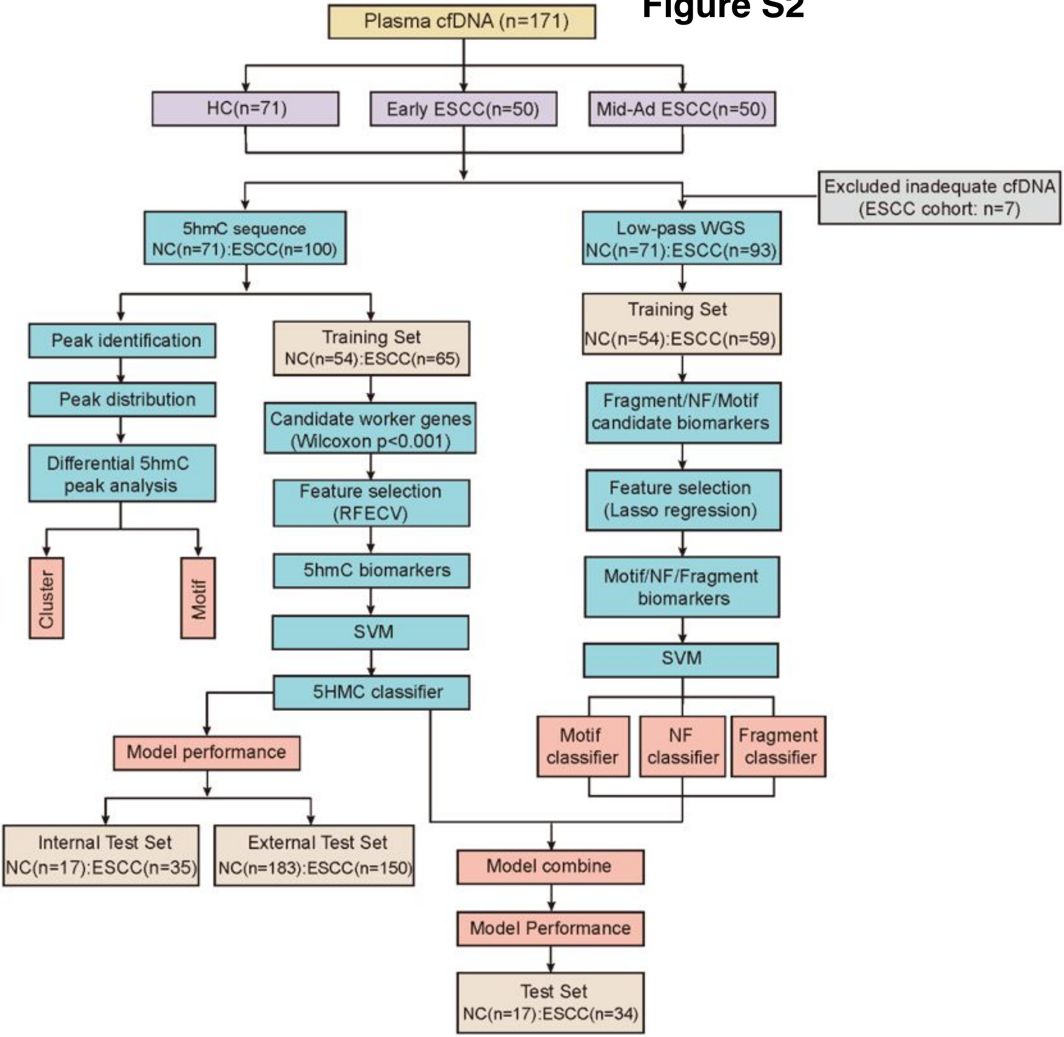

Plasma cfDNA (n=171)
HC(n=71)
Early ESCC(n=50)
Mid-Ad ESCC(n=50)
Excluded inadequate cfDNA (ESCC cohort: n=7)
5hmC sequence NC(n=71):ESCC(n=100)
Low-pass WGS NC(n=71):ESCC(n=93)
Peak identification
Peak distribution
Differential 5hmC peak analysis
Cluster
Motif
Training Set NC(n=54):ESCC(n=65)
Candidate worker genes (Wilcoxon p<0.001)
Feature selection (RFECV)
5hmC biomarkers
SVM
5HMC classifier
Model performance
Internal Test Set NC(n=17):ESCC(n=35)
External Test Set NC(n=183):ESCC(n=150)
Training Set NC(n=54):ESCC(n=59)
Fragment/NF/Motif candidate biomarkers
Feature selection (Lasso regression)
Motif/NF/Fragment biomarkers
SVM
Motif classifier
NF classifier
Fragment classifier
Model combine
Model Performance
Test Set NC(n=17):ESCC(n=34)

Figure S3

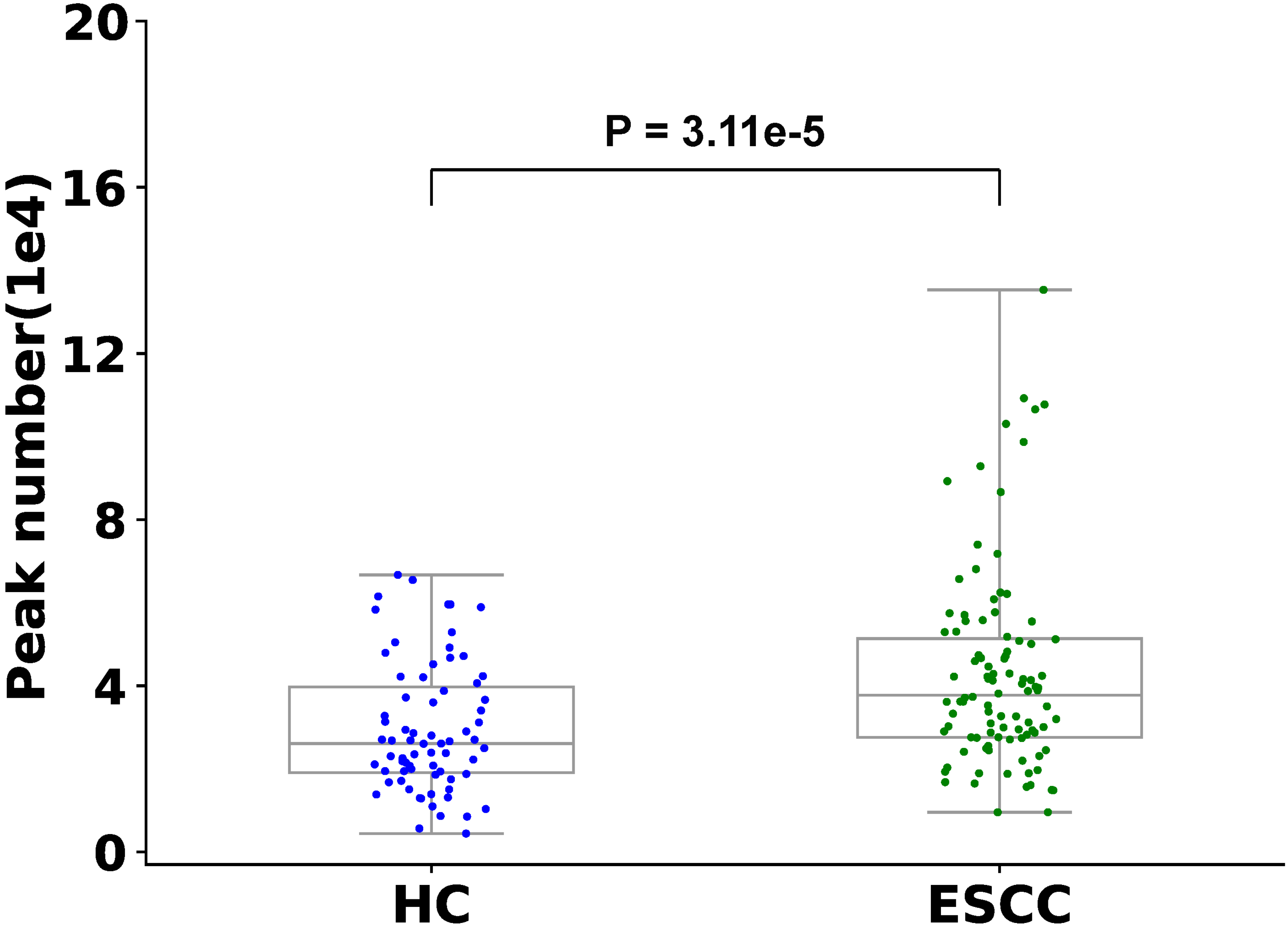

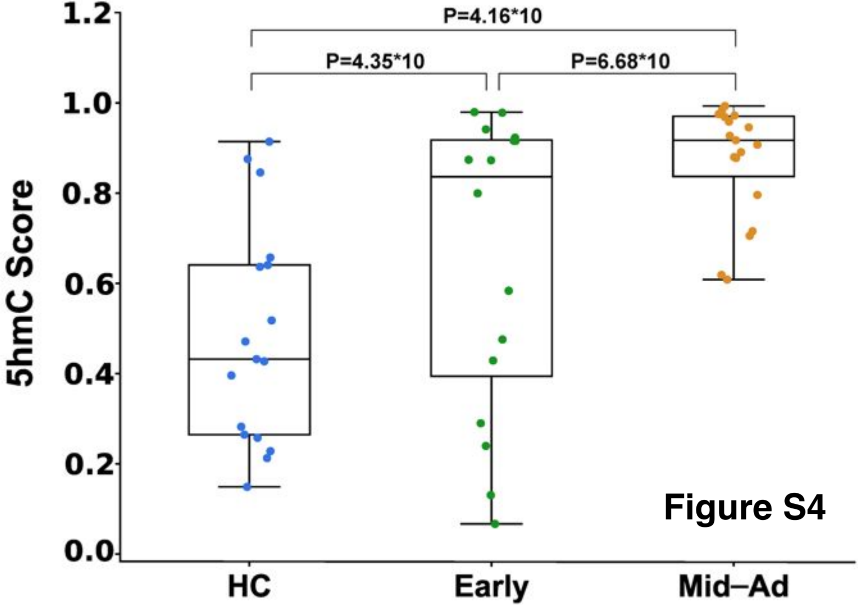
P=4.16*10
P=4.35*10
P=6.68*10
5hmC Score
1.2
1.0
0.8
0.6
0.4
0.2
0.0
HC
Early
Mid−Ad

Figure S4

Figure S5

A

Motif-test		
**Classes**	**Actual_ESCC**	**Actual_HC**
**Predicted_ESCC**	25	3
**Predicted_HC**	9	14
**Se/Sp**	73.5%	82.4%

NF-test		
**Classes**	**Actual_ESCC**	**Actual_HC**
**Predicted_ESCC**	31	5
**Predicted_HC**	3	12
**Se/Sp**	91.2%	70.6%

Fragment-test		
**Classes**	**Actual_ESCC**	**Actual_HC**
**Predicted_ESCC**	27	3
**Predicted_HC**	7	14
**Se/Sp**	79.4%	82.4%

5hmC-test		
**Classes**	**Actual_ESCC**	**Actual_HC**
**Predicted_ESCC**	26	3
**Predicted_HC**	8	14
**Se/Sp**	76.5%	82.4%

B

Stage	5hmC classifier accuracy	Stage	Combined classifier accuracy
P0 (n-6)	33.3% (2/6)	P0 (n=5)	80.0% (4/5)
I (n=10)	70.0% (7/10)	I (n=10)	80.0% (8/10)
II (n9)	88.9% (8/9)	II (n=9)	88.9% (8/9)
III (n=8)	87.5% (7/8)	III (n=8)	75.0% (6/8)
IV (n-2)	100% (2/2)	Iv (n=2)	100% (2/2)
**Total (n=35)**	**74.3% (26/35)**	**Total (n=34)**	**82.4% (28/34)**
